# Intersections of Fibrodysplasia Ossificans Progressiva and Traumatic Heterotopic Ossification

**DOI:** 10.3390/biom14030349

**Published:** 2024-03-14

**Authors:** Conan Juan, Alec C. Bancroft, Ji Hae Choi, Johanna H. Nunez, Chase A. Pagani, Yen-Sheng Lin, Edward C. Hsiao, Benjamin Levi

**Affiliations:** 1 Center for Organogenesis, Regeneration, and Trauma, Department of Surgery, University of Texas Southwestern Medical Center, Dallas, TX 75390, USAjihae.choi@utsouthwestern.edu (J.H.C.);; 2 Baylor College of Medicine, Houston, TX 77030, USA; 3 Department of Orthopaedic Surgery, University of Texas Southwestern Medical Center, Dallas, TX 75390, USA; yen-sheng.lin@utsouthwestern.edu; 4 Division of Endocrinology and Metabolism, Department of Medicine, the Institute for Human Genetics, and the Program in Craniofacial Biology, University of California San Francisco Medical Center, San Francisco, CA 94143, USA; edward.hsiao@ucsf.edu

**Keywords:** heterotopic ossification, fibrodysplasia ossificans progressiva, ectopic bone, ACVR2, ALK2, trauma

## Abstract

Heterotopic ossification (HO) is a debilitating pathology where ectopic bone develops in areas of soft tissue. HO can develop as a consequence of traumatic insult or as a result of dysregulated osteogenic signaling, as in the case of the orphan disease fibrodysplasia ossificans progressiva (FOP). Traumatic HO (tHO) formation is mediated by the complex interplay of signaling between progenitor, inflammatory, and nerve cells, among others, making it a challenging process to understand. Research into the pathogenesis of genetically mediated HO (gHO) in FOP has established a pathway involving uninhibited activin-like kinase 2 receptor (ALK2) signaling that leads to downstream osteogenesis. Current methods of diagnosis and treatment lag behind pre-mature HO detection and progressive HO accumulation, resulting in irreversible decreases in range of motion and chronic pain for patients. As such, it is necessary to draw on advancements made in the study of tHO and gHO to better diagnose, comprehend, prevent, and treat both.

## 1. Introduction

Heterotopic ossification (HO) is the development of ectopic bone in regions of soft tissue, including joint spaces, tendons, and muscles around the appendicular joints. It is a known consequence of traumatic events, such as burns or blast fractures, but can also occur as a complication of surgical procedures like total hip arthroplasty [1], underlying inflammatory conditions like dermatomyositis [2], or neurologic injury like traumatic brain injury [3]. Studies conducted to better understand the mechanism behind traumatic heterotopic ossification (tHO) have revealed tHO to be the result of a complex signaling interchange between diverse cell types, including mesenchymal stem cells (MSCs), inflammatory cells, and nerves, among others. The heterogeneous cell population present in the environment in which tHO develops has made clinical advancements in preventing and treating tHO difficult.

In contrast to tHO, genetically mediated heterotopic ossification (gHO) includes congenital diseases, such as fibrodysplasia ossificans progressiva (FOP) and progressive osseous heteroplasia, where ectopic bone can form independently from trauma. Here, we focus on FOP as a specific form of gHO. FOP is an ultra-rare disease with a prevalence of 0.88 per million in the US, leading to its designation as an orphan disease recognized by the National Organization for Rare Disorders [4]. Given the disabling and irreversible nature of gHO following FOP inflammatory flare-ups, studies have been conducted to improve our understanding of the disease. Research on FOP has led to the development of drugs that are being evaluated in clinical trials. Given the strides that have been made in understanding both tHO and gHO in FOP, the goal of this review is to connect ideas between the two fields to show how research of either condition can inform our ability to diagnose, understand, prevent, and treat both forms of HO. 

## 2. Clinical Picture of tHO and FOP

The clinical presentations of tHO and FOP have overlapping features. The mature stage of HO manifests clinically as hard, palpable lesions of ectopic bone found in areas of soft tissue throughout the body. Traumatic HO development results in severe pain, swelling, warmth, and debilitating decreases in range of motion (ROM) at the affected site [5,6] and can occur after burns blast injuries, amputations, and deep orthopedic surgeries like total hip arthroplasty [1,5]. The locations of these bony lesions can determine the impact that HO has on patients’ overall quality of life. For example, HO in the head and neck can lead to difficulty with day-to-day tasks, including oral hygiene, eating, swallowing, and speaking [7,8]. HO in the knee most often contributes to loss of flexion at the joint [9], along with other patellofemoral complications, including instability and patellofemoral tracking disorder [10]. Similarly, HO that occurs after hip arthroplasty results in decreased ROM and, in more severe cases, can lead to sciatic nerve irritation and femur dislocation [10]. HO in other sites markedly increases mortality, as seen with thoracic HO, which compromises the airway. Mass effect from HO development in the thoracic cavity results in thoracic insufficiency syndrome, ultimately leading to hypoxemia, pneumonia, and heart failure [8,11]. In addition, masses near the surface of the body can increase the risk of skin breakdown and pressure sores [12,13].

FOP leads to the development of early onset HO through genetically mediated mechanisms. This genetic form of HO presents with similar clinical symptoms as non-genetic tHO—pain, swelling, and decreased range of motion—but with a much more pervasive and progressive presentation. In addition to the increased occurrence of these bony lesions at a young age, nearly all patients with FOP present with congenital bilateral hallux valgus deformities [8], along with a malformed great toe due to structural abnormalities in the first phalanx and metatarsal [5]. Osteochondromas at the proximal medial tibia and spinal manifestations, including spinal fusions at the levels of C2 through C7 and scoliosis, are also common in FOP. Individuals with FOP may also have elongated vertebrae, shortened femoral necks, soft-tissue swellings at the scalp and other sites, along with thumb malformations [8]. The presence of skeletal malformations (especially in the great toes), migratory swellings, and HO lesions should suggest the need for further work-up to evaluate for and diagnose FOP quickly, before any biopsies or procedures are performed, as these can trigger further HO formation [14].

## 3. Current Understanding of Mechanisms behind tHO and FOP

The formation of heterotopic ossification in both tHO and FOP relies on signaling between numerous ligands and receptors. This combinatorial diversity contributes to the different phenotypes of HO that are observed across the clinical spectrum and may explain why some tissue regions are seemingly more predisposed to either of the different types of HO (e.g., appendicular skeletal lesions with tHO and axial skeletal lesions with FOP) [5]. The complexity of these interactions has made understanding HO regulation a challenge. Interestingly, many of the mechanisms found to govern tHO and gHO formation appear to be shared. This is particularly true of the TGF-β superfamily of ligands and their associated receptors, discussed in further detail below.

### 3.1. Transforming Growth Factor Beta (TGF-β) Superfamily Signaling

The TGF-β superfamily is a very large signaling family that includes type I and II receptors that bind to various ligands, such as TGF-β, bone morphogenic protein (BMPs), activins, inhibins, and growth factor-β. Ligands show mixed affinity for type I and II receptors located on the cell membrane, but ultimately, both receptors are recruited to create tetrameric complexes that include two type I and two type II receptors [15]. Type II receptors have constitutive kinase activity and phosphorylate type I receptors once complexed by ligand binding [16]. Once phosphorylated, type I receptors can phosphorylate different receptor-activated Smad proteins (Smad 1,2,3,5,8) located in the cytosol, which are responsible for intracellular signaling. In Smad-dependent signaling, TGF-β and activin ligands signal through Smad2/3 complexes, whereas BMP ligands signal through Smad1/5/8 complexes. Both Smad2/3 and Smad1/5/8 complexes associate with Smad4 for translocation into the nucleus to regulate gene transcription. TGF-β superfamily intracellular signaling can also occur through Smad-independent (non-canonical) pathways, such as ERK [17], TAK1 [18], p38 MAP [19], and PI3K/AKT [20]. In summary, TGF-β signaling occurs through the formation of tetrameric complexes, which include type I and II receptors that can signal through Smad-dependent pathways (typically Smad2/3 for TGF-β and Activins, or Smad1/5/8 for BMPs) or Smad-independent pathways.

#### 3.1.1. TGF-β Ligands Regulating Traumatic HO

There are three isoforms of TGF-β ligands: TGF-β1, TGF-β2, and TGF-β3. TGF-β ligands bind to TGF-βR1 (ALK5), TGF-βR2, or ALK1 and complexes initiating intracellular signaling via the phosphorylation of Smad2/3 complexes. Phosphorylated Smad2/3 further complexes with Smad4 to translocate to the nucleus to regulate gene expression. TGF-β ligands have been shown to promote MSC recruitment [21,22] and proliferation (via β-catenin) [23,24]. The independent inhibition of TGF-β1, TGF-β2, or TGF-β3 produces skeleton malformations, demonstrating that all isoforms play a role in regulating normal bone development [25,26,27]. Studies that inhibited or knocked out TGF-βR1 or 2 have affected bone development [28,29], further supporting the role of TGF-β signaling in bone development. TGF-β signaling has been associated with the early stages of chondrocyte and osteoblast differentiation [30]. Interestingly, TGF-β signaling has been shown to inhibit the later stages of osteoblast maturation and bone matrix formation, as measured by the decreased expression of *Runx2* and *Ocn* [29,31,32,33]. Excessive TGF-β signaling has been implicated in diseases that create weaker bone structures, such as osteogenesis imperfecta [34] and osteoporosis [35]. Collectively, these studies suggest that TGF-β signaling plays an important role in promoting early stages of chondrogenesis and osteogenesis while inhibiting later stages.

TGF-β1, specifically derived from macrophages, has been shown to be an important regulator in tHO formation [36,37]. TGF-β2 and TGF-β3 have not been thoroughly studied in the context of tHO formation. While TGF-β signaling exists within MSCs, it can also occur in other cell populations. Interestingly, the deletion of TGF-βR1/ALK5 in macrophages inhibited tHO formation, whereas deletion in zeugopod-specific MSCs showed no effect on tHO formation [38]. Together, this suggests that TGF-β signaling in macrophages, rather than MSCs, plays a more important role in tHO formation.

#### 3.1.2. BMP Ligands Regulating Traumatic HO

The BMP signaling family currently includes 15 ligands that can induce signaling among several type 1 and 2 receptors that can form complexes with one another [39]. Given so many potential combinations, BMP signaling is a highly complex process that researchers are continuing to investigate to better understand. In general, BMP ligands bind to type 1 (ALK1, ALK2/ACVR1, ALK3/BMPRIA, ALK4/ACVR1B, ALK5/TGFBRI, and ALK6/BMPRIB) and type 2 (BMPRII, ActRII, and ActRIIB) complexes, initiating intracellular signaling [39]. Smad1/5/8 signaling is typically associated with the type I receptors except for ALK4 and 5. BMP2, 4, 6, 7, and 9, have been shown to regulate osteogenesis [40,41,42,43] and chondrogenesis [44] through Smad1/5/8 signaling. In addition, BMP2 and 4 have been shown to promote chondrogenesis through the regulation of SOX9 expression [44,45,46,47]. These BMPs affect different steps in osteoblast maturation as well as bone matrix formation by upregulating RUNX2, OSX/SP7, OCN, and ALP [48,49,50,51,52,53,54,55,56].

BMP2, 4, 6, 7, and 9 have been shown to promote tHO formation [57,58,59,60,61,62]. In one study, the individual knockout of type I receptors—ALK2, 3, and 6—resulted in no statistically significant differences in tHO formation. Although not statistically significant (*p*-value = 0.09), the knockout of ALK2 only resulted in a reduction in ectopic bone formation, suggesting that ALK2 may still play a role in tHO. Interestingly, knockout of both ALK2 and 3 resulted in a statistically significant reduction in tHO formation [63], suggesting that multiple BMP ligands and receptors may regulate signaling. Other studies have investigated the efficacy of anti-ALK2 and anti-ALK2/3 antibodies, which have resulted in statistically significant reductions in tHO formation [64,65]. While pharmacological targeting of ALK2 and 3 resulted in less tHO formation, it also delayed wound healing and led to methicillin-resistant Staphylococcus metastatic infections, suggesting the isolated targeting of ALK 2 and 3 is a poor clinical therapy [63,64]. However, targeting BMP ligands via a soluble ALK3-Fc antibody resulted in reduced tHO formation with no notable side effects [63,64,65]. Together, these data suggest that multiple BMP ligands and receptors regulate tHO formation, and therapies targeting BMP ligands could provide a clinically effective method to reduce tHO with minimal adverse side effects.

TGF-β activated kinase 1 (TAK1) is a member of the mitogen-activated protein kinase family that can also regulate tHO formation [66,67]. TAK1 has been shown to activate Smad1/5/8 and smad-independent signaling via p38/JNK/ERK MAP kinase [68,69]. TAK1 has been shown to promote the expression of chondrogenic (SOX9 [70]) and osteogenic (OCN, ALP, RUNX2 [18]) genes. In addition to affecting Smad-dependent signaling, TAK1 regulates the stabilization and nuclear localization of YAP/TAZ [71], which have also been shown to regulate tHO formation [72].

### 3.2. Genetic Mutations in ALK2/ACVR1 Causing gHO in Fibrodysplasia Ossificans Progressiva (FOP)

FOP is associated with gain-of-function mutations, leading to an overactive activin-like kinase 2 (ALK2) receptor, also called activin A receptor type 1 (*ACVR1*). ALK2 is a serine/threonine kinase receptor classified as a bone morphogenic protein (BMP) type I receptor and is a member of the larger transforming growth factor beta superfamily. ALK2 is ubiquitously expressed throughout the body, and the dysregulation of this receptor leads to downstream effects in multiple tissue types [73].

#### ALK2 Signaling Is Dysregulated in FOP

**Figure 1:**
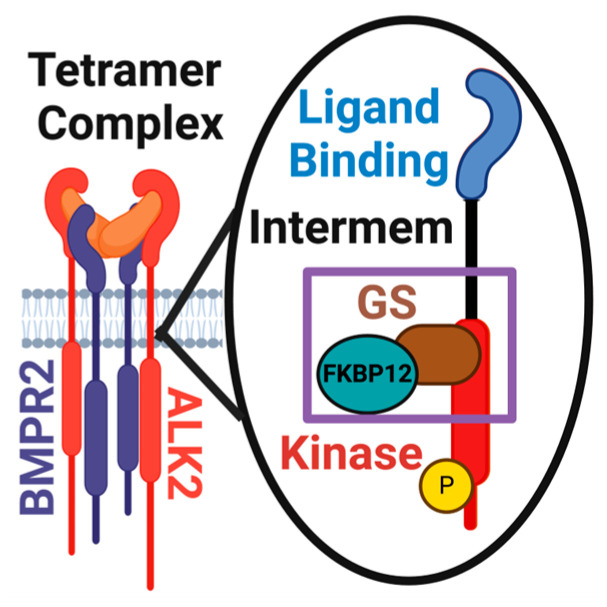
**ALK2 Structure.** Tetramer complex of BMPR2 and ALK2. Individual domains of ALK2 are shown and labeled. The purple box indicates region of ALK2 that is altered in FOP.

The ALK2 receptor is composed of five main domains: a signaling peptide, a ligand binding region, an intermembrane region, a glycine–serine (GS) rich region, and a protein kinase region (Figure 1) [74]. Upon binding to a ligand, ALK2 associates with a BMP type 2 receptor (BMPR2), such that there are two ALK2 receptors and two BMPR2 receptors, resulting in a tetrameric complex. The association with BMPR2 receptors allows ALK2 to undergo a conformational change at the GS intracellular region, allowing for the release of FKBP12. The release of FKBP12 serves as a regulator for the initiation of ALK2 kinase activity and intracellular signaling [75,76,77].

Smad-dependent signaling pathways, particularly Smad1/5/8, are associated with osteogenic differentiation through ALK2. When ALK2 binds to a BMP ligand, intracellular signaling is initiated through the phosphorylation of the protein complex Smad1/5/8. After phosphorylation, Smad1/5/8 complexes with Smad4, allowing for translocation into the nucleus to serve as a transcription factor. Through the Smad1/5/8 pathway, ALK2 serves as an essential mediator in gastrulation [78], neuropathic pain [79], inflammation [80], chondrogenesis [81,82], and osteogenesis [83]. When ALK2 binds to an activin ligand, intracellular signaling is initiated through the phosphorylation of receptor-mediated Smad2/3. In summary, normal ALK2 intracellular signaling has both constitutive and ligand-dependent activity. The binding of the ALK2 receptor with BMPs initiates a Smad1/5/8 pathway that promotes BMP pathway activation, whereas activins normally activate the Smad2/3 pathway.

**Figure 2:**
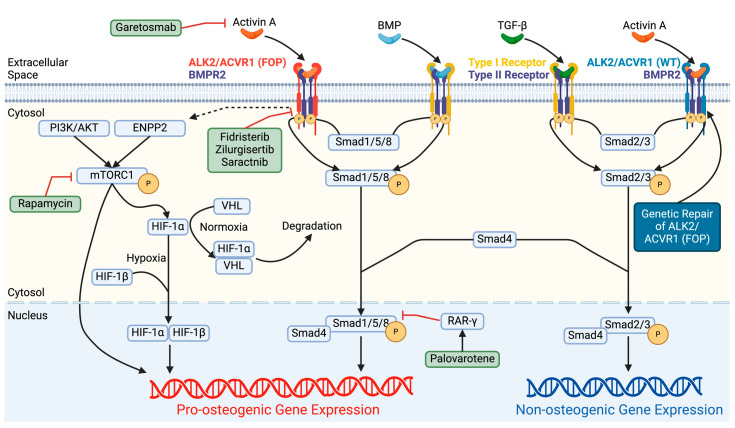
**Dysregulation of activin A-ALK2 signaling in FOP.** Mutation in the GS region of ALK2 leads to an inappropriate increase in BMP-regulated signaling in response to activin A, leading to pro-osteogenic signaling in skeletal stem cell-like cells. Green boxes include therapies in ongoing clinical trials. Dark blue box indicates therapies targeted at genetic repair.

In patients with FOP, ALK2 has a mutation that gives it a novel and abnormal ability to initiate pro-osteogenic Smad1/5/8 signaling upon binding by activin A. This effectively allows ALK2-expressing cells to misinterpret activin A as a BMP. Approximately 95% of patients with FOP have an activating mutation that results in a histidine replacing an arginine at codon 206 (R206H) within the GS region of the *ACVR1/ALK2* gene [84]. This R206H mutation creates a conformational change in the GS region of ALK2 such that FKBP12 has a reduced ability to bind to this region and inhibit the activity of the kinase region [85]. Therefore, this mutation leads to increased and uncontrolled ALK2 signaling (Figure 2). The R206H mutation results in increased responsiveness to BMP2, 4, 7, 9, and 10, as well as novel responsiveness to BMP15 and activins A, AB, AC, and B [86]. Activin ligands form dimers—both homodimers (activin A and B) or heterodimers (activin AB and AC). Using a monoclonal antibody specific to the activin A unit, ectopic bone formation significantly decreased in mouse models with a R206H mutation in their ALK2 receptor, suggesting that activin A is the primary ligand driving abnormal osteogenesis in MSCs [86]. Further experimentation has shown that activin A binding to the R206H mutated receptor induces intracellular Smad1/5/8 signaling [86,87]. The Smad1/5/8 complex associates with Smad4 to translocate into the nucleus. Once in the nucleus, this Smad1/4/5/8 complex can be inhibited by retinoic acid receptor-γ, which inhibits ectopic bone formation [88]. In summary, the R206H mutation in ALK2 is common in FOP patients and results in an increased and uncontrolled pro-osteogenic Smad1/5/8 (BMP) signaling induced by activin A.

### 3.3. Understanding of FOP Mechanism Informs the Future of tHO Studies

#### 3.3.1. Activin A and ALK2

As previously discussed, it is common in FOP to see increased BMP (Smad1/5/8) signaling through a neofunction in the ALK2-R206H receptor upon binding with an activin A ligand. Ectopic bone formation in FOP has been reported following spontaneous inflammatory flare-ups, injury, intramuscular immunization, viral infection, or overuse [89]. Given the inflammatory response that precedes ectopic bone formation in FOP [90], studies have been conducted to better understand the role of the immune system in FOP. Previous research has demonstrated that mast cells and macrophages play an essential role in the progression of gHO in FOP [91]. Interestingly, macrophages isolated from FOP patients demonstrated increased activin A expression and pro-inflammatory cytokine (IL-1a, TNF, IL-6, IFN-γ) release when compared to baseline M1 macrophages [92]. This suggests that macrophages play a key role in sustaining a pro-inflammatory response and serve as a source of activin A for gHO induction. Therefore, therapies aimed at inhibiting the immune response during FOP flare-ups may reduce activin A secretion from macrophages and inhibit ectopic bone formation. While targeting the immune system may reduce macrophage-derived activin A, other cell populations can also contribute to activin A secretion at sites of ectopic bone formation. In FOP mouse models, single-cell RNA sequencing revealed fibroblasts with increased expression of *Inhba* (activin A) following tamoxifen-induced gluteal muscle injury, suggesting that fibroblasts may be another source of activin A in regions of gHO formation in FOP [65]. Still, it remains possible that activin A may come from sources outside of the tissue region that forms ectopic bone. A recent study demonstrated that serum activin A levels were not statistically elevated in untreated FOP patients compared to healthy control subjects either during FOP flare-ups or remission [93]. Together, previous research suggests that the major source of activin A contributing to gHO formation in FOP is from local cell populations, including macrophages and fibroblasts. Given the complex nature of FOP flare-ups and large variation in anatomical regions where gHO forms, further investigation is needed to better understand the spatial and temporal contexts of activin A expression in cell populations at the site of ectopic bone formation in FOP.

Studies have investigated the efficacy of targeting activin A and ALK2 receptors in FOP. Anti-activin A antibodies have been shown to be effective in inhibiting Smad1/5/8 signaling and ectopic bone formation [86,94]. One study demonstrated that the overexpression of wild-type ALK2 served as an effective way to inhibit ectopic bone formation in FOP mice [95]. Increasing the number of wild-type ALK2 receptors may reduce the opportunity for activin A to bind to mutated ALK2-R206H receptors and decrease the subsequent Smad1/5/8 signaling that has been implicated in ectopic bone formation. This suggests that the targeting of activin A via an antibody (i.e., garetosomab) may be an effective therapy to reduce gHO formation in FOP. With regard to targeting ALK2, one study demonstrated that the treatment of FOP mice with an anti-ALK2 antibody unexpectedly resulted in the activation of ALK2-R206H receptors, unlike wild-type ALK2 receptors, which resulted in more ectopic bone formation [94]. Still, in another study and mouse model, saracatinib was used to target ALK2 and effectively reduced ectopic bone formation while not impacting neonatal growth [96]. While targeting activin A or ALK2 may serve as promising therapies to prevent ectopic bone formation in FOP, ALK2 is expressed in various cells throughout the body [97], and activin A has a role in proper skeletal development and regulating immune system functioning [98,99,100]. Additionally, given that multiple different mutations can lead to the development of FOP [84], future studies should investigate genetic repair mechanisms aimed at restoring normal function to mutated ALK2 receptors. In summary, targeting activin A and ALK2 may serve as effective therapies against gHO formation in FOP, but future research should focus on repairing the gene itself to restore proper function to the mutated ALK2 receptor.

Given the importance of activin A and ALK2 in FOP, researchers have studied both in the context of tHO formation. Single-cell RNA sequencing experiments used on cells isolated from a subcutaneous BMP-implant mouse model revealed mesenchymal progenitor cells (MPCs) and, to a lesser extent, macrophages expressing *Inhba* (activin A) as the major cell populations in tHO lesions [101]. In a burn/tenotomy (B/T) mouse model, *Inhba* was primarily expressed by pericytes and smooth muscle cells following injury [65]. Together, these studies reveal that the cell populations contributing to activin A are different between tHO and FOP. Currently, there are conflicting data on the impact of activin A in tHO formation. In experiments that used a subcutaneous or intramuscular BMP implant mouse model, anti-activin A antibodies were shown to significantly but not completely inhibit tHO formation [101]. Therefore, while activin A may contribute to tHO formation in these models, there are likely other mechanisms that regulate its formation. Interestingly, in the B/T model, the use of an anti-activin A antibody was not effective in inhibiting tHO formation [65]. It is important to note that the BMP-implant and B/T models induce ectopic bone formation in different anatomical regions with different and unique cell populations (i.e., tenocytes in the B/T model). Coupled with the differences in the efficacy of anti-activin A antibodies, it is likely that different mechanisms contribute to tHO formation in different anatomical regions. With respect to ALK2, studies using the burn/tenotomy mouse model revealed that anti-ALK2 antibodies significantly, but not completely, inhibit tHO formation [65]. This further supports the idea that there are mechanisms outside of activin A-ALK2 signaling that contribute to tHO formation. Together, this suggests that while activin A-ALK2 signaling plays a role in driving tHO formation, there are likely other mechanisms driving tHO formation that are different from FOP.

#### 3.3.2. Hypoxia

Hypoxic conditions are present at injury sites following trauma and have also been reported in FOP lesions [102] that are destined to form bone. Cytosolic hypoxia-inducible factors (HIFs) have the ability to regulate gene expression based on oxygen levels. Under normoxic conditions, HIF-1α complexes with VHL and is degraded [103]. Under hypoxic conditions, HIF-1α is stabilized and able to translocate into the nucleus, where it complexes with HIF-1β to regulate gene expression [104,105]. It has been demonstrated that pre-chondrogenic FOP lesions are positive for HIF-1α, confirming that hypoxic conditions are present with inflammation and early stages of tissue remodeling [102]. In vitro and in vivo studies with HIF-1α knockout have demonstrated a reduction in pSmad1/5/8 signaling cells, suggesting that hypoxic conditions promote BMP signaling [102]. It was further demonstrated that in vivo knockout or pharmacological inhibition (imatinib, apigenin, PX478, and rapamycin) of HIF-1α in FOP mice resulted in a significant reduction in ectopic bone formation [102,106]. While these drugs inhibit HIF-1α, only PX478 directly targets HIF-1α. It has been demonstrated that activin A-ALK2 signaling in FOP mice promotes the mammalian target of rapamycin-1 (mTORC1) signaling, which is crucial in regulating chondrogenesis and ectopic bone formation in FOP [107]. Given that rapamycin directly targets mTORC1 and inhibits HIF-1α, these findings suggest that mTORC1 is upstream of HIF-1α [108,109]. Further investigations have found that mTORC1 is downstream of ENPP2 and the PI3KT/AKT axis. The expression of *ENPP2*, a gene that encodes for the secretory enzyme autotaxin that produces lysophospholipid acid [110], was upregulated in MSCs isolated from FOP mice following activin A induction, suggesting a possible mechanism for increased mTOR signaling. It is still unclear what the exact mechanism connecting activin A-ALK2 signaling with mTOR signaling in FOP; therefore, future investigations are needed. Still, in one study, the stimulation of FOP cells with activin A demonstrated increased mTOR signaling but no change in HIF-1α expression, indicating that HIF-1α may be a mechanism independent of activin A [111]. Together, these studies demonstrate that HIF-1α under hypoxic conditions contributes to ectopic bone formation in FOP, and further studies are needed to understand the underlying mechanism.

In the context of tHO, HIF-1α has also been found to be upregulated following injury in osteogenic regions. Similar to FOP studies, in vivo knockout, knockdown, or pharmacological inhibition (directly by PX478 and indirectly by rapamycin) of HIF-1α in tHO mice resulted in a significant reduction in ectopic bone formation [106,112]. Recent studies have shown that vascular endothelial growth factor A (VEGFA), an angiogenic protein whose expression is modulated by HIF-1α, is upregulated in MSCs as well as macrophages following injury, suggesting that hypoxia may modulate tHO formation through cell populations other than MSCs [36,113]. In another recent study, HIF-1α was shown to influence ectopic bone formation by promoting M2 macrophage phenotypes and osteoclast formation following intramuscular implantation of osteoinductive material [114]. Given that inflammation and hypoxia tend to exist concurrently, more studies are warranted to better understand MSC-specific mechanisms and other immune cell population contributions in FOP and tHO.

## 4. Identification and Diagnosis of HO

### 4.1. Traumatic HO

Diagnosis of tHO relies on a combination of clinical picture, serum markers, and radiographic findings. Neurogenic HO (nHO), a subset of tHO, can occur after spinal cord injury (SCI), resulting in significant morbidity and compromised quality of life [115]. The timing of tHO, whether neurogenic in nature or otherwise, starts within 1–3 weeks of contractures around the appendicular skeleton. However, current X-ray techniques are not able to reliably detect HO prior to 6 weeks [5,6]. Some prognostic factors used in clinical measures are assessments of clinical signs (e.g., contractures), serum biomarkers (e.g., alkaline phosphatase [ALP], C-reactive protein [CRP], and creatine phosphokinase [CPK]), radiographic imaging assessments (e.g., X-ray, computer tomography), and questionnaires (e.g., International Spinal Cord Injury Musculoskeletal Basic Data Set). The timelines of current clinical diagnostic tests for tHO following SCI are listed in Table 1.

**Figure 3:**
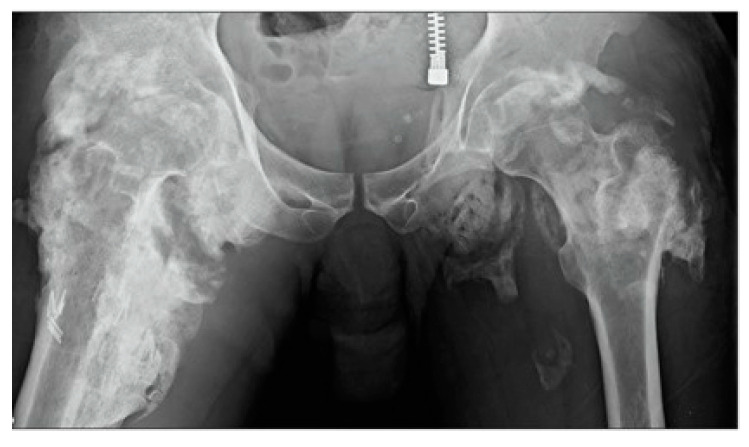
Radiographic-based diagnostic imaging represented massive bilateral peri-articular HO in an immobilized patient with SCI [118].

The current classification schemes for assessing tHO include the planar projection of mineralization using a four-level radiological classification [116] or checkerboard-like patterns within the muscle regions observed via computer tomography (CT). These radiographic-based assessments involve global estimates of the degree of soft tissue mineralization [117], but they do not effectively detect pre-mature bone formation, limiting its use to monitoring the progress of tHO and implementing early-stage diagnosis and timed therapeutic strategies at the bedside. Figure 3 shows an example of radiographic imaging from our prior case with massive bilateral HO in an immobilized patient with SCI [118]. Furthermore, tHO is associated with elevated serum ALP, CPK, C-reactive protein (CRP), erythrocyte sedimentation rate (ESR), and prostaglandin E2 (PGE2) [119], which are reliable and sensitive indicators of the tHO formation process following spinal cord injury. Elevated levels of serum CPK are an indicator of HO severity, while the stabilization of ALP is contradictory with HO maturation [120,121]. Table 2 shows the lab test characteristics of tHO and non-tHO patients. Despite the fact that the differences observed in serum biomarker levels in these studies suggest their ability to aid in HO diagnosis, further exploration into their clinical use will be important for solidifying our understanding of their utility.

The current standard of care only detects HO after irreversible functional deficits have already occurred. Imaging of tHO lesions like those shown in Figure 3 often leads to their misdiagnosis as bone tumors, obviating the need not only to detect but also to diagnose tHO [122]. Radiographic modalities lead to inconsistent and inaccurate diagnoses and fail to guide treatment initiation or duration. These limitations have kept clinicians from establishing precision/personalized medicine approaches to SCI-induced HO. Although triple-phase bone scans detect HO activity before calcification becomes apparent on plain X-ray and CT imaging (Table 1), this technology requires an injected radioactive tracer and has yet to be effectively developed for clinical use [123]. Traumatic HO can also be confirmed with diagnostic ultrasound [124] and magnetic resonance imaging [125]. These modalities can be used as a screening tool if there is a high index of suspicion of tHO but should then be confirmed by one of the tests listed below (Table 3).

### 4.2. Fibrodysplasia Ossificans Progessiva (FOP)

Currently, the diagnosis of FOP is made by clinical presentation (presence of 1st digit malformations like toe malformations, with migratory swellings/inflammation) combined with genetic testing for *ACVR1* mutations. However, our ability to diagnose whether new HO formation will occur in FOP remains poor. As such, advancements made in diagnostic modalities traditionally used for tHO may be applicable to gHO, allowing for earlier recognition of HO development in FOP patients. Furthermore, individuals with early onset recurrent HO, bilateral congenital hallux valgus malformations, and other features suggestive of FOP can undergo confirmatory genetic testing in the form of single-gene testing targeting the gain of function mutations in *ACVR1* [8]. In addition to testing for mutations in *ACVR1,* clinicians can also implicate other genes whose aberrant activity causes clinical pictures that overlap with FOP using more comprehensive genomic exome sequencing or a skeletal dysplasia panel, which tests for mutations in ~20 genes, including *EXT1/2*, *GNAS*, *PTPN11*, and *ROR2* [8].

## 5. Progenitor Cell Populations in tHO and FOP

Genetic HO formation in FOP is attributed to a mutation in the *ACVR1* gene that disrupts cell signaling pathways involved in bone formation while preserving typical endochondral ossification in bones [84,126]. These disruptions induce alterations in surrounding microenvironmental factors that trigger the development of chondral and osteogenic cell lineages, ultimately resulting in ectopic bone formation and changes in cell fate determination [87,127,128]. The primary cells impacted by these environmental changes are progenitor cells, which have remarkable potential to differentiate into specialized cell types such as lymphocytes, myocytes, osteoblasts, osteoclasts, and adipocytes. Their capability to differentiate has led progenitor cells to play a pivotal role in tissue injury and subsequent HO formation.

Progenitor cells are pluripotent stem cell descendants that have the ability for self-renewal and expansion, particularly in response to trauma, disease, and aging. Identification of progenitor populations involved in FOP has given insight into the underlying mechanisms of and potential cell-specific therapeutic approaches for this disease. The exploration of progenitor populations has been significantly facilitated by using animal models. In particular, the development of the *Acvr1* knock-in mouse line, representative of human FOP, has been instrumental in advancing FOP research [86,129]. Subsequent studies using animal models and various Cre drivers have developed researchers’ ability to assess progenitor cells’ osteogenic capacity and provided insight into the diverse progenitor cell lineages directly involved in HO formation (Table 4).

### 5.1. Hematopoietic Stem Cells

Hematopoietic stem cells (HSCs) are quiescent cells in the bone marrow, which are capable of multi-lineage differentiation into all blood cell types and self-renewal through hematopoiesis [141]. There are limited research studies investigating HSCs’ involvement in HO, and those that are available have provided conflicting evidence of HSC involvement. Several clinical case studies have shown evidence of trilineage hematopoiesis in excised tHO bone [131,142,143,144,145]. Although these case studies indicate the presence and involvement of HSCs in the HO site, there is insufficient evidence to indicate that these cells are involved in the process of osteogenesis in HO formation. Some research studies have suggested that HSCs are involved in the regulation of osteoblasts for bone formation [146,147,148]. However, one study found that HSCs do not contribute to osteogenesis in tHO formation [149]. Further research is necessary to confirm and elucidate the mechanism by which HSCs contribute to HO formation.

While HSCs’ direct involvement in HO formation is yet to be understood, there is evidence highlighting the intricate roles of hematopoietic lineage cells derived from HSCs in this process. HSCs have the ability to differentiate into immune cells from both the myeloid and lymphoid lineages [150]. While inflammation is a normal physiological response to injury, FOP studies have suggested that chronic inflammation induced by the disease engenders a prolonged and hyperactive immune system, which is regulated through hematopoietic lineage cells, which promotes HO development. Myeloid lineage cells can further differentiate into granulocytes and monocytes, while lymphoid lineage cells can differentiate into T cells, B cells, and natural killer (NK) cells [151,152]. There has been evidence showing HSC involvement through lymphocyte infiltration and mast cell destruction of skeletal muscle during the early stages of FOP flare-ups [130,153,154,155]. Subsequent studies have shown that monocytes are required to trigger HO formation through the release of osteogenic factors in transgenic mice [156]. Additionally, it has been found that fibroproliferative tissues developed after injury show active immune and mast cells, giving insight into the involvement of immune cells in tissue remodeling during HO formation [129,153].

### 5.2. Endothelial Progenitor Cells

Using lineage tracing and transgenic mice, endothelial progenitor cells expressing Tie2 have been found to be major contributors to and present in all stages of HO formation. Several studies have shown Tie2-expressing cells contributing to the fibroproliferative, chondrogenic, and osteogenic stages of HO formation [132,133]. However, Tie2 is not specific to endothelial cells; the gene is also expressed in platelet-derived growth factor α (PDGFRα) receptors and fibro/adipogenic progenitors, potentially indicative of mesenchymal and muscle origin instead [157]. Recent studies have suggested an endothelial-to-mesenchymal transition (EndMT) in HO formation. This transition was revealed to be caused by mutations with ALK2 activation in FOP. EndMT was further validated with the observation of endothelial markers in the chondrocytes and osteoblasts of HO lesions [133].

Additionally, endothelial progenitor cells are directly involved in angiogenesis, giving rise to blood vessel sprouting. In FOP patients, there is an increase in vascular endothelial growth factor (VEGF) following inflammatory stimuli compared to control. Increased expression of VEGF promotes the infiltration of angiogenesis, driving HO formation [158]. There have been further studies describing vascular leakage and edema in HO lesions from patients with FOP, further supporting endothelial and angiogenic interplay in FOP [159].

### 5.3. Mesenchymal Stem Cells

Mesenchymal stem cells are pluripotent cells that have the potential to differentiate into osteogenic, chondrogenic, adipogenic, and myogenic lineages [160,161]. HO formed in FOP involves the replacement of cartilage with bone, orchestrated by osteoclasts derived from HSCs and osteoblasts of the MSC lineage [135]. While MSCs have the potential to differentiate into chondrogenic and osteogenic lineages alone, the environmental changes induced by FOP promote MSC differentiation into chondrocytes, osteoblasts, and osteocytes through osteoblastic maturation [162,163]. Specifically, the *ACVR1* mutation in FOP leads to the aberrant activation of BMP signaling in response to activin A, a normal ligand involved in the TGF-β signaling pathway, which induces the chondrogenesis of MSCs [87].

### 5.4. Muscle Stem Cells

Muscle injury is known to trigger tHO formation and exacerbate disease flare-ups in FOP, suggesting the involvement of aberrant skeletal muscle regeneration in HO formation. Muscle stem cells, also known as muscle satellite cells, reside in skeletal muscle tissue and are responsible for muscle repair and regeneration following muscle injury [164,165]. In vitro studies have shown that satellite cells exhibit osteogenic activity in response to BMPs [166,167]. Consequently, a recent investigation explored the impact of *ACVR1* mutation on skeletal muscle repair by collecting human FOP satellite cells. These cells exhibited deficiencies in muscle repair and regeneration capabilities through increased ECM and osteogenic markers compared to control satellite cells. This suggests that satellite cells contribute to HO through reprogramming towards an osteogenic environment [136]. In a subsequent in vivo study using a FOP mouse model, they found that muscle injury induced muscle tissue to reprogram towards chondrogenesis in FOP mice but not in wild-type mice [168]. These studies suggest that the *ACVR1* mutation in FOP induces muscle stem cells to reprogram towards an osteogenic fate. While muscular injury can lead satellite cells down an osteogenic fate in FOP, it does not always lead to tHO formation in the context of trauma. Further research is needed to understand the role and underlying mechanism of satellite cells, specifically in tHO.

### 5.5. Fibro/Adipogenic Progenitor Cells

Fibro/adipogenic progenitor cells (FAPs) are mesenchymal stromal cells that reside in the skeletal muscle, distinct from satellite cells. They are key regulators of skeletal muscle regeneration and contribute to the pathologic differentiation of skeletal muscle into fibroblasts and adipocytes [169,170,171]. Previous research has demonstrated that FAPs undergo reprogramming towards an endochondral lineage [138,172]. In a FOP (ACVR1-R206H) mouse model, osteogenic differentiation was induced in FAPs through the activin ligand activation of BMP signaling in both injury-induced and spontaneous HO models [137]. It was further revealed that FAPs impacted the myogenic activity of satellite cells, which suggests that the coordination between FAPs and satellite cells is important for HO formation in FOP [172].

Identifying progenitor cell populations involved in gHO formation in FOP has allowed us to understand the underlying pathophysiology of this disease. It has also provided us with greater insight into understanding tHO formation mechanisms and opportunities for potential therapeutics as they both undergo endochondral ossification. Through the identification of progenitor cell populations in FOP, several studies have found potential therapeutics and have proven their efficacy in mouse models [173]. While there is substantial evidence of overlapping progenitor populations such as HSCs, EPCs, and MSCs, there is also evidence of distinct progenitor populations between gHO in FOP and tHO.

### 5.6. Tendon Stem/Progenitor Cells

Tendon stem/progenitor cells (TSPCs) reside in the peritenon of the tendon [174]. Using two different injury animal models, it was recently discovered that TSPCs contributed to both cartilage and bone formation in tHO formation through osteochondral differentiation following trauma [140].

The identification of progenitor cells associated with gHO in FOP has advanced our understanding of the underlying mechanism of this disease. In FOP, the development of an accurate genetic model resembling human FOP has been paramount in identifying the pathological mechanisms behind this disease. Despite the importance of various animal lineages and injury models to current research, the translatability of conclusions drawn from these models to clinical applications remains unclear. The exact mechanisms behind tHO are debated and less understood. Further investigation is needed to validate a robust model and identify pathological HO formation, with the aim of discovering specific cell-therapeutic targets for the prevention and treatment of HO.

## 6. Inflammatory Control of HO

Inflammation plays a key role in both the gHO found in FOP and tHO, and the immune system is a vital component of both normal and abnormal bone formation. In normal bone, osteoclasts are thought to derive from monocyte precursors [175]. Osteoblasts, while differentiating from MSCs, have been shown to show significant impairment in macrophage-deficient mice [176]. Additionally, osteal tissue-resident macrophages, also known as osteomacs, are known to play a role in anabolic bone formation [177]. While vital to normal ossification, the inflammatory process may play a more crucial role in the abnormal formation of bone, specifically in the case of HO. Studying the inflammatory cells and pathways in FOP is instrumental in guiding and understanding HO in both its genetic and traumatic forms.

FOP is associated with inflammatory lesions that occur after flare-ups that can lead to significant HO formation. As previously discussed, FOP is primarily caused by activating mutations in *ACVR1*, which lead to abnormal BMP signaling in response to activin A [126]. However, *ACVR1* mutations leading to FOP do not explain the flare-ups or inflammatory nature of gHO, suggesting the involvement of other inflammatory factors and processes [178].

Multiple immune cell types have been showed to be involved in gHO formation [129]. In early fibroproliferative lesions from FOP patients, researchers found BMP4 upregulation in lymphoblastoid cell lines [179]. Similar studies in mouse models have shown that hematopoietic stem cells play a role in the early inflammation phases of BMP4-induced HO [180]. Using mouse hematopoietic stem cell transplants, researchers found that transplantation of normal bone marrow alone did not attenuate FOP progression; however, immunosuppression of these mice led to decreased HO formation [180]. Other studies have shown the involvement of monocytes, macrophages, and mast cells at the sites of abnormal bone formation in FOP [90,129]. When HO lesions were investigated in FOP patients, mast cells were present at every stage of development, with early FOP lesions showing the presence of perivascular inflammatory infiltrates [91,102,153]. In a conditional mouse knockout model of mast cells, the HO volume was reduced by 50% in mice with *ACVR1* mutations, revealing an essential role of mast cells in HO [91]. Furthermore, monocyte and macrophage lineages also appear to play a significant role in the inflammatory process of FOP, as BMP receptors are robustly expressed on monocytes and tissue macrophages involved in HO formation [102,181]. Monocytes isolated from FOP patients have also shown evidence of increased DNAM-1 expression, which plays a role in monocyte migration, leading to the thought that monocytes play a role in the early activation of FOP flare-ups [182].

In addition to these inflammatory cells, human blood samples have demonstrated significantly increased cytokine levels and inflammatory pathways in FOP patients. When monocytes collected from FOP patients were stimulated with lipopolysaccharide (LPS), they showed the prolonged activation of NF-κB, suggesting its role in FOP inflammation [37]. These and other findings have shown that *ACVR1* activity causes a pro-inflammatory state through increased NF-κB [183]. Additionally, transforming growth factor beta (TGF-β), a cytokine released by monocytes and macrophages, is increased in FOP patients and has been shown to attenuate HO formation in FOP mouse models when systemically suppressed [37,118]. This implicates TGF-β as a potential inducer and promoter of HO [37]. As TGF-β is linked to tissue repair macrophages, it also implicates myeloid cells’ role in FOP HO formation during early inflammatory phases.

In a similar manner to FOP, tHO has shown to be at least in part driven by inflammatory processes, many of which overlap with FOP inflammatory mechanisms. Like FOP patients, individuals with tHO have shown evidence of increased immune cell presence as well as increased inflammatory cytokines such as IL-3, IL-6, IL-10, and MCP-1 following blast and penetrating combat injuries [184,185,186,187]. Burn/tenotomy HO mouse models have also shown early increased levels of IL-6 and IL-1α in mice that form HO [187]. Recent studies have shown that the NF-κB signaling pathway plays an important role in tHO; when the NF-κB cascade was blocked, HO formation significantly decreased [188]. Similar to TGF-β pathways implicated in FOP, a recent study in a burn mouse model revealed that TGF-β1-producing macrophages are associated with HO, and a systemic reduction in macrophage-produced TGF-β levels helped to ameliorate HO [186]. Overall, these studies illustrate that both gHO and tHO are heavily influenced by inflammatory cells and pathways. While inflammatory cells such as macrophages, mast cells, and adaptive immune cells play roles in the development of HO, how they trigger the activation of HO formation remains to be fully elucidated. Continuing to study the link between inflammation in FOP and tHO can lead to synergistic advancements of knowledge in both fields and hopefully lead to new potential treatments for all patients with HO.

## 7. Nervous System Involvement in HO

FOP and tHO both cause increased levels of pain. Patients suffering from FOP experience moderate to severe pain at baseline as well as during flare-ups [189,190]. Traumatic HO patients often endure pain at the injury or surgical site during ectopic bone formation as well as months and years after HO has matured [3,145,191]. HO tissue excised from patients has been previously shown to be highly innervated, which may contribute to the pain experienced by patients [192]. Because of these reports, researchers have investigated the role of the peripheral nervous system in ectopic bone formation.

Following a burn/tenotomy model of HO, peptidergic and sympathetic autonomic nerves innervate the tendon injury site. The inhibition of nerve signaling, either by sciatic neurectomy or pharmacologic blockage of nerve growth factor (NGF) or its receptor TrkA, reduced neural ingrowth and HO formation [192]. Neurectomy ultimately reduced HO formation by altering chondrogenic differentiation [192]. Interestingly, nerves recruit new blood vessels through the secretion of pro-angiogenic factors, similar to the role of nerves in endochondral ossification during normal bone development [193,194]. BMP2 induction models of HO, where BMP2 is injected into muscle, resulting in HO development, show peripheral nerve involvement [195]. The neurogenesis found in these models was associated with increased levels of mast cell infiltration and degranulation at the BMP2 induction site. Cromolyn inhibition of this degranulation reduced HO formation, suggesting a neuroinflammatory role in HO [195].

Neurogenic forms of tHO have been documented after insults to the central nervous system. Cerebral vascular accidents have been implicated in the development of nHO [196,197], and both traumatic brain injury (TBI) and SCI have been reported to significantly increase the risk of nHO [3,198,199]. Additionally, patients suffering from TBI with concurrent fractures report increased fracture healing rates [200], suggesting a pro-osteogenic effect following nervous system injury. While there is limited research on the effect of TBI or SCI on ectopic bone formation, elegant studies have described the effect of the adrenergic nervous system on macrophage phenotype switching to M2, which increases osteogenesis [201]. Further work will need to be performed to elucidate whether these factors or others are at play in nHO.

The peripheral nervous system provides pro-osteogenic niches in tHO, recruiting critical factors for chondrogenic and osteogenic differentiation. The neurogenesis following injury may contribute to the increased pain experienced by patients; however, it remains unclear whether reducing neural ingrowth also reduces HO-related pain. Further, some patients with FOP exhibit significant neurologic phenotype with heightened sensitivity to pain, suggesting nervous system involvement in FOP that still needs to be elucidated [79]. Future animal studies of FOP, tHO, and nHO should include functional tests, such as von Frey and algometer testing, to better understand how pain is modulated with treatments.

## 8. Current Therapeutics for HO

### 8.1. Traumatic HO

Treatment for tHO once it has matured is limited. Surgical removal represents a controversial but potentially effective option. The early resection of lesions within the first year of diagnosis has positive results, with relatively low rates of recurrence [202,203]. However, in many cases, the risk of recurrence, along with the technical difficulty of fully removing tHO from anatomical locations like the thoracic cavity, make surgery a less optimal solution for some patients [5]. As a result, many of the remaining therapies relating to tHO are supportive or prophylactic in nature.

While complete removal and cure of tHO remains a challenge, supportive measures can be taken to alleviate patients’ symptoms. Physical therapy (PT) is known to improve pain and range of motion with other disabling conditions [204] and can be considered for patients with deficits in range of motion due to tHO. However, PT has not been well-studied in the context of HO, and conflicting opinions on the impact of PT on HO exacerbation exist within the PT community [205]. As such, PT’s impact on tHO and its symptoms represents a worthwhile avenue for exploration.

The prevention of tHO formation in high-risk individuals (i.e., individuals undergoing planned procedures like total hip arthroplasty) can be achieved using certain therapeutics. While these methods have demonstrated prophylactic efficacy, they each come with their own set of drawbacks. Non-steroidal anti-inflammatory drugs (NSAIDs), like indomethacin, have been demonstrated to prevent tHO formation after hip replacement [206]. However, their effectiveness in preventing tHO after other surgeries is inconsistent [207], and they come with their own host of gastrointestinal and renal side effects. Radiation therapy courses attenuate HO formation if initiated within 48 h of hip surgery but come at the risk of secondary malignancy [208]. Bisphosphonates, like etidronate, have historically been used for tHO prophylaxis but are cost-prohibitive and do not exhibit significantly better outcomes when compared to NSAIDs [209].

### 8.2. Genetic HO

Currently, there are no curative treatments for FOP. Standard-of-care therapy remains focused on supportive measures, including the judicious use of glucocorticoids and NSAIDs within 24–48 h of a flare-up to decrease the excessive inflammation present in early FOP lesions [89]. However, these therapies are not particularly effective in preventing HO, and they do not mitigate the progressive nature of this disease. Furthermore, there are notable side effects with long-term corticosteroid use, making this a less favorable drug for chronic treatment. Mast-cell inhibitors and leukotriene inhibitors are also often used on a chronic basis to empirically address the inflammatory aspect of early FOP lesions [210,211]. Bisphosphonates are occasionally used for refractory flare-ups that do not respond to glucocorticoids; however, concrete clinical data for these treatments are sparse [211]. Avoidance of trauma and injury, which would, in turn, reduce inflammation, remains the mainstay of therapy. Surgical resection, which can be used in some non-genetic causes of heterotopic ossification (e.g., trauma, burns, spinal cord injuries, and hip surgery), is contraindicated in patients with FOP as it can induce inflammation and trigger a cascade of unrelenting, excessive bone formation at both the surgical site and at distant locations [89,212,213,214].

**Figure 4:**
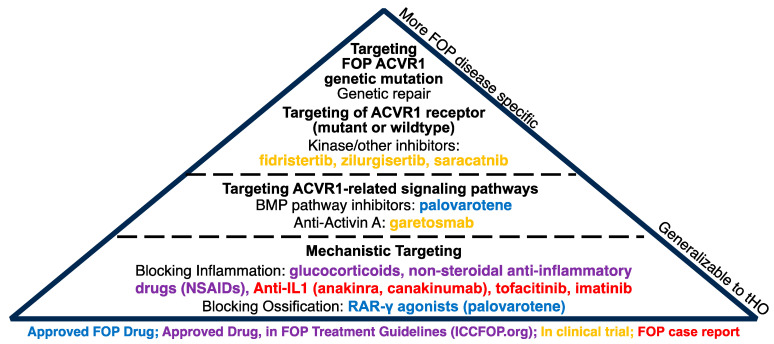
**Potential Therapeutic Strategies for FOP**. Illustration of potential therapeutics for treatment of FOP, organized from FOP-specific (top) to more generalizable treatments (bottom) that should be studied for potent use in tHO. Therapeutics shown vary in strength of evidence: approved FOP drug (blue), approved drug in FOP treatment guidelines (purple), in clinical trial (yellow), and FOP case report (red).

Recent clinical discoveries and research have identified several potential therapeutic options for managing new HO formation in FOP (Figure 4). Multiple compounds are currently being evaluated in clinical trials. These include palovarotene (NCT05027802) [215,216], a recently approved retinoic acid receptor-γ agonist that blocks BMP signaling and the conversion of cartilage to bone; garetosmab (NCT05394116) [217,218], an anti-activin A antibody that decreases the neo-ligand signaling induced by the ACVR1^R206H^ mutation; rapamycin [219] (https://center6.umin.ac.jp/cgi-open-bin/ctr/ctr_view.cgi?recptno=R000032495, accessed on 4 September 2023), an immunosuppressant with anti-proliferative properties; and several kinase inhibitors directed against *ACVR1*, including zilurgisertib (INCB000928, NCT05090891), fidristertib (IPN60130, NCT05039515), and saracatnib (NCT04307953).

In addition, several medications have been considered for off-label use based on case reports showing potential benefits in FOP. A number of these are immunomodulators, including canakinumab [220], imatinib [220], and tofacitinib [221]. These therapeutic directions target different stages of HO, and some may be more specific to FOP. However, drugs that have more mechanistic targets rather than FOP-specific targets, such as palovarotene and immune modulators, may also find potential benefits for non-genetic forms of HO and warrant further study. Examples include potential uses of anti-IL1 therapies in nHO [222] and NSAIDs for prophylaxis against tHO after total hip arthroplasties [223,224]. Further, while radiation therapy can be used for tHO prophylaxis, it is not used in patients with FOP due to the risks of inducing further muscle inflammation [225] and triggering subsequent HO.

## 9. Conclusions

Heterotopic ossification is a debilitating disease process that can be mediated by both genetic and non-genetic mechanisms. The development of HO relies on the interplay of signaling within a heterogeneous environment. Genetically mediated forms of HO found in FOP follow a known pathway involving a mutation in *ACVR1*, leading to dysregulated ALK2 signaling down a pro-osteogenic pathway. The identification of this signaling cascade has allowed for the development of drug therapies like palovarotene and garetosmab. While this signaling cascade has been well-established, the growth of ectopic bone in gHO and tHO remains a complex and elusive process involving progenitor, inflammatory, and nerve cells. The goal of this review was to provide insights into the current understanding of how each of these cell populations contributes to HO pathology. Progenitor cell research has identified discrete populations that differentiate directly into HO, as well as create an environment conducive to osteogenic development. Inflammatory cells, like macrophages, mast cells, and adaptive immune cells, have been implicated in the development of both gHO and tHO. Further, the study of HO in the context of its neurological environment is still developing, and with further advancements in this field, we will have a more comprehensive view of HO development within both genetic and traumatic contexts. While no reliable, curative treatments exist for either tHO or gHO once it has formed, clinical studies on compounds for the prophylaxis and prevention of HO development are in progress. The development of non-invasive, point-of-care (POC) diagnostic modalities that can pinpoint the early stages of tHO and nHO to detect early HO formation and progression following traumatic events is desperately needed for an improved clinical decision support system for HO management. For gHO, targeted therapies at *ACVR1* and downstream signaling are being assessed. More generalizable therapeutics, like NSAIDs for prophylaxis, have shown potential outside of FOP with tHO and will need to be further evaluated. Overall, while significant strides are still needed for the clinical treatment of both traumatic and genetic HO, recent advancements have improved our ability to diagnose, understand, prevent, and treat them both.

## Figures and Tables

**Table 1:** **Timelines of traumatic HO detection methods following spinal cord injury**. (CPK = creatine phosphokinase; CRP = C-reactive protein; PGE2 = prostaglandin E2; ALP = alkaline phosphatase).

Parameter	Postinjury Time
Transient ↓ in serum Ca^2+^	1 week
↑ CPK and CRP	1 week
↑ Urinary PGE2	1 week
↑ serum ALP level	2 weeks
+ve triple phase bone scan	3 weeks
+ve radiograph	4–6 weeks

**Table 2:** **Serum lab test characteristics of tHO and non-tHO patients**.

Lab Tests	tHO Group	Non-tHO Group
Alkaline phosphatase (ALP)	>130 U/L	20–130 U/L
C-reactive protein (CRP)	10–100 mg/L	<1.0 mg/L
Creatine phosphokinase (CPK)	1–10 mg/L	<1 mg/L
Erythrocyte sedimentation rate (ESR)	16–100 mm/h	<15 mm/h

**Table 3:** **Advantages, risks, and limitations of current clinically available diagnostic modalities and available research tools for HO detection**.

	X-ray	CT Scan	Triple Phase Bone Scan	MRI	Diagnostic Ultrasound
Advantages	Cost-effective, reliable, and sensitive for HO diagnosis	Comprehensive, reliable, and sensitive for HO diagnosis	Early detection before calcification	Comprehensive and reliable to indicate HO formation	Portable, sensitive, and cost-effective to indicate HO formation
Risks	Light ionization radiation exposure	Moderate ionization radiation exposure	Contrast agent required and moderate radiation exposure	Not applicable to those with implant, pacemaker, and intracranial aneurysm clips	No measurable risks but may introduce non-measurable bias
Limitations	Qualitative and unable to detect pre-HO soft tissue mineralization	Qualitative Limited access in some regions.	Limited access in regions with less resourceful healthcare.	Qualitative, expensive, and not commonly used for HO early detection. Limited access in some regions.	Subjective and qualitative measures
Timing of HO diagnosis after SCI	4–6 weeks	4–6 weeks	2–3 weeks	2–4 weeks	1–2 weeks

**Table 4:** **Progenitor cell types in heterotopic ossification (HO)**.

Progenitor Cell Type	Disease	Findings	Model (Lineage Tracing Marker)	Study
Hematopoietic Stem Cells (HSCs)	FOP/ tHO	HSCs give rise to cells that contribute to early inflammatory and fibroproliferative stage of HO Hematopoiesis evidence found in patient excised tHO	Human	Gannon et al. (1998) [130] Davis et al. (2013) [131]
Endothelial Progenitor Cells (EPCs)	FOP/tHO	Tie2+ EPCs contribute to every stage of HO formation Chondrocytes and osteoblasts express endothelial markers, suggesting endothelial-to-mesenchymal transition (EndMT) in FOP-HO lesionsAngiogenesis drives HO formation in FOP; inhibition of angiogenesis attenuates HO progression in tHO	Mouse (Tie2-Cre)	Lounev et al. (2009) [132] Medici et al. (2010) [133] Lin et al. (2022) [134]
Mesenchymal Stem Cells (MSCs)	FOP	MSCs increase osteochondrogenesis in FOP Nfatc1+ cells induce spontaneous HO lesions with increased osteogenic potential	Human Mouse (Nfatc1-Cre)	Hino et al. (2015) [87] Agarwal et al. (2015) [135]
Muscle Stem Cells	FOP	Muscle stem cells exhibit enhanced osteogenic and chondrogenic fate following muscular injury in FOP	Human	Barruet et al. (2021) [136]
Fibro/Adipogenic Progenitor Cells (FAP)	FOP	Activin A drives osteogenesis in FAPs, leading to spontaneous gHO formation	Mouse (MyoD-iCre/Tie2-Cre)	Lees-Shepard et al.(2018) [137]
Tendon Stem/Progenitor Cells	FOP/tHO	Scx+ cells induce spontaneous HO formation and are capable of chondrogenic and osteogenic differentiation involved in both gHO and tHO Tppp3+ cells contribute to chondrogenesis and osteogenesis after trauma	Mouse (Scx-Cre/Tppp3+)	Dey et al. (2016) [138] Agarwal et al. (2017) [139] Yea et al. (2023) [140]

## References

[1] Ranganathan K., Loder S., Agarwal S., Wong V.W., Forsberg J., Davis T.A., Wang S., James A.W., Levi B. (2015). Heterotopic Ossification: Basic-Science Principles and Clinical Correlates. J. Bone Jt. Surg..

[2] Eckardt J.J., Ivins J.C., Perry H.O., Unni K.K. (1981). Osteosarcoma arising in heterotopic ossification of dermatomyositis: Case report and review of the literature. Cancer.

[3] Cipriano C.A., Pill S.G., Keenan M.A. (2009). Heterotopic Ossification Following Traumatic Brain Injury and Spinal Cord Injury. J. Am. Acad. Orthop. Surg..

[4] Pignolo R.J., Hsiao E.C., Baujat G., Lapidus D., Sherman A., Kaplan F.S. (2021). Prevalence of fibrodysplasia ossificans progressiva (FOP) in the United States: Estimate from three treatment centers and a patient organization. Orphanet J. Rare Dis..

[5] Hwang C.D., Pagani C.A., Nunez J.H., Cherief M., Qin Q., Gomez-Salazar M., Kadaikal B., Kang H., Chowdary A.R., Patel N. (2022). Contemporary perspectives on heterotopic ossification. J. Clin. Investig..

[6] Sullivan M.P., Torres S.J., Mehta S., Ahn J. (2013). Heterotopic ossification after central nervous system trauma. Bone Jt. Res..

[7] Schoenmaker T., Bouchankouk A.D., Özkan S., Gilijamse M., Bouvy-Berends E., Netelenbos C., Lobbezoo F., Eekhoff E.M.W., de Vries T.J. (2022). Limitations of Jaw Movement in Fibrodysplasia Ossificans Progressiva: A Review. Front. Med..

[8] Akesson L.S., Savarirayan R., Adam M.P., Feldman J., Mirzaa G.M., Pagon R.A., Wallace S.E., Bean L.J.H., Gripp K.W., Amemiya A. (2023). Fibrodysplasia Ossificans Progressiva. GeneReviews.

[9] Furia J.P., Pellegrini V.D. (1995). Heterotopic ossification following primary total knee arthroplasty. J. Arthroplast..

[10] Iorio R., Healy W.L. (2002). Heterotopic Ossification After Hip and Knee Arthroplasty: Risk Factors, Prevention, and Treatment. J. Am. Acad. Orthop. Surg..

[11] Wentworth K.L., Bigay K., Chan T.V., Ho J.P., Morales B.M., Connor J., Brooks E., Salamat M.S., Sanchez H.C., Wool G. (2018). Clinical-pathological correlations in three patients with fibrodysplasia ossificans progressiva. Bone.

[12] Potter B.K., Burns T.C., Lacap A.P., Granville R.R., Gajewski D. (2006). Heterotopic Ossification in the Residual Limbs of Traumatic and Combat-Related Amputees. J. Am. Acad. Orthop. Surg..

[13] Appelt E.A., Kenkel J.M., Ballard J.R., Lopez J.A., Anthony T., Castillo T. (2005). Preoperative Embolization of Heterotopic Ossification for the Treatment of a Recalcitrant Pressure Sore. Plast. Reconstr. Surg..

[14] Kaplan F.S., Al Mukaddam M., Baujat G., Brown M., Cali A., Cho T.J., Crowe C., De Cunto C., Delai P., Diecidue R. The Medical Management of Fibrodysplasia Ossificans Progressiva: Current Treatment Considerations. https://www.iccfop.org/dvlp/wp-content/uploads/2022/05/guidelines-updated-May-2022.pdf.

[15] Schmierer B., Hill C.S. (2007). TGFβ–SMAD signal transduction: Molecular specificity and functional flexibility. Nat. Rev. Mol. Cell Biol..

[16] Wrighton K.H., Lin X., Feng X.-H. (2009). Phospho-control of TGF-β superfamily signaling. Cell Res..

[17] Xie L., Law B.K., Chytil A.M., Brown K.A., Aakre M.E., Moses H.L. (2004). Activation of the Erk Pathway Is Required for TGF-β1-Induced EMT In Vitro. Neoplasia.

[18] Yang H., Guo Y., Wang D., Yang X., Ha C. (2018). Effect of TAK1 on osteogenic differentiation of mesenchymal stem cells by regulating BMP-2 via Wnt/β-catenin and MAPK pathway. Organogenesis.

[19] Yu L., Hébert M.C., Zhang Y.E. (2002). TGF-beta receptor-activated p38 MAP kinase mediates Smad-independent TGF-beta responses. EMBO J..

[20] Bakin A.V., Tomlinson A.K., Bhowmick N.A., Moses H.L., Arteaga C.L. (2000). Phosphatidylinositol 3-Kinase Function Is Required for Transforming Growth Factor β-mediated Epithelial to Mesenchymal Transition and Cell Migration. J. Biol. Chem..

[21] Tang Y., Wu X., Lei W., Pang L., Wan C., Shi Z., Zhao L., Nagy T.R., Peng X., Hu J. (2009). TGF-β1–induced migration of bone mesenchymal stem cells couples bone resorption with formation. Nat. Med..

[22] Chen Y., Wurtz T., Wang C., Kuo Y., Yang K.D., Huang H., Wang F. (2004). Recruitment of mesenchymal stem cells and expression of TGF-β1 and VEGF in the early stage of shock wave-promoted bone regeneration of segmental defect in rats. J. Orthop. Res..

[23] Zhou S. (2011). TGF-β regulates β-catenin signaling and osteoblast differentiation in human mesenchymal stem cells. J. Cell. Biochem..

[24] Jian H., Shen X., Liu I., Semenov M., He X., Wang X.-F. (2006). Smad3-dependent nuclear translocation of β-catenin is required for TGF-β1-induced proliferation of bone marrow-derived adult human mesenchymal stem cells. Genes Dev..

[25] Kaartinen V., Voncken J.W., Shuler C., Warburton D., Bu D., Heisterkamp N., Groffen J. (1995). Abnormal lung development and cleft palate in mice lacking TGF–β3 indicates defects of epithelial–mesenchymal interaction. Nat. Genet..

[26] Sanford L.P., Ormsby I., Groot A.C.G.-D., Sariola H., Friedman R., Boivin G.P., Cardell E.L., Doetschman T. (1997). TGFβ2 knockout mice have multiple developmental defects that are non-overlapping with other TGFβ knockout phenotypes. Development.

[27] Kulkarni A.B., Huh C.G., Becker D., Geiser A., Lyght M., Flanders K.C., Roberts A.B., Sporn M.B., Ward J.M., Karlsson S. (1993). Transforming growth factor beta 1 null mutation in mice causes excessive inflammatory response and early death. Proc. Natl. Acad. Sci. USA.

[28] Seo H.-S., Serra R. (2007). Deletion of Tgfbr2 in Prx1-cre expressing mesenchyme results in defects in development of the long bones and joints. Dev. Biol..

[29] Mohammad K.S., Chen C.G., Balooch G., Stebbins E., McKenna C.R., Davis H., Niewolna M., Peng X.H., Nguyen D.H.N., Ionova-Martin S.S. (2009). Pharmacologic Inhibition of the TGF-β Type I Receptor Kinase Has Anabolic and Anti-Catabolic Effects on Bone. PLoS ONE.

[30] Rosen D.M., Stempien S.A., Thompson A.Y., Seyedin S.M. (1988). Transforming growth factor-beta modulates the expression of osteoblast and chondroblast phenotypes in vitro. J. Cell. Physiol..

[31] Kang J.S., Alliston T., Delston R., Derynck R. (2005). Repression of Runx2 function by TGF-β through recruitment of class II histone deacetylases by Smad3. EMBO J..

[32] Alliston T., Choy L., Ducy P., Karsenty G., Derynck R. (2001). TGF-beta-induced repression of CBFA1 by Smad3 decreases cbfa1 and osteocalcin expression and inhibits osteoblast differentiation. EMBO J..

[33] Maeda S., Hayashi M., Komiya S., Imamura T., Miyazono K. (2004). Endogenous TGF-β signaling suppresses maturation of osteoblastic mesenchymal cells. EMBO J..

[34] Grafe I., Yang T., Alexander S., Homan E.P., Lietman C., Jiang M.M., Bertin T., Munivez E., Chen Y., Dawson B. (2014). Excessive transforming growth factor-β signaling is a common mechanism in osteogenesis imperfecta. Nat. Med..

[35] Erlebacher A., Derynck R. (1996). Increased expression of TGF-beta 2 in osteoblasts results in an osteoporosis-like phenotype. J. Cell Biol..

[36] Tu B., Li J., Sun Z., Zhang T., Liu H., Yuan F., Fan C. (2022). Macrophage-Derived TGF-β and VEGF Promote the Progression of Trauma-Induced Heterotopic Ossification. Inflammation.

[37] Wang X., Li F., Xie L., Crane J., Zhen G., Mishina Y., Deng R., Gao B., Chen H., Liu S. (2018). Inhibition of overactive TGF-β attenuates progression of heterotopic ossification in mice. Nat. Commun..

[38] Patel N.K., Nunez J.H., Sorkin M., Marini S., Pagani C.A., Strong A.L., Hwang C.D., Li S., Padmanabhan K.R., Kumar R. (2022). Macrophage TGF-β signaling is critical for wound healing with heterotopic ossification after trauma. J. Clin. Investig..

[39] Katagiri T., Watabe T. (2016). Bone Morphogenetic Proteins. Cold Spring Harb. Perspect. Biol..

[40] Kang Q., Song W.-X., Luo Q., Tang N., Luo J., Luo X., Chen J., Bi Y., He B.-C., Park J.K. (2009). A Comprehensive Analysis of the Dual Roles of BMPs in Regulating Adipogenic and Osteogenic Differentiation of Mesenchymal Progenitor Cells. Stem Cells Dev..

[41] Bandyopadhyay A., Tsuji K., Cox K., Harfe B.D., Rosen V., Tabin C.J. (2006). Genetic Analysis of the Roles of BMP2, BMP4, and BMP7 in Limb Patterning and Skeletogenesis. PLoS Genet..

[42] Ebisawa T., Tada K., Kitajima I., Tojo K., Sampath T.K., Kawabata M., Miyazono K., Imamura T. (1999). Characterization of bone morphogenetic protein-6 signaling pathways in osteoblast differentiation. J. Cell Sci..

[43] Kemmis C.M., Vahdati A., Weiss H.E., Wagner D.R. (2010). Bone morphogenetic protein 6 drives both osteogenesis and chondrogenesis in murine adipose-derived mesenchymal cells depending on culture conditions. Biochem. Biophys. Res. Commun..

[44] Zhou N., Li Q., Lin X., Hu N., Liao J.-Y., Lin L.-B., Zhao C., Hu Z.-M., Liang X., Xu W. (2016). BMP2 induces chondrogenic differentiation, osteogenic differentiation and endochondral ossification in stem cells. Cell Tissue Res..

[45] Semba I., Nonaka K., Takahashi I., Takahashi K., Dashner R., Shum L., Nuckolls G.H., Slavkin H.C. (2000). Positionally-dependent chondrogenesis induced by BMP4 is co-regulated by sox9 and msx2. Dev. Dyn..

[46] Pan Q., Yu Y., Chen Q., Li C., Wu H., Wan Y., Ma J., Sun F. (2008). Sox9, a key transcription factor of bone morphogenetic protein-2-induced chondrogenesis, is activated through BMP pathway and a CCAAT box in the proximal promoter. J. Cell. Physiol..

[47] Zehentner B.K., Dony C., Burtscher H. (1999). The Transcription Factor Sox9 Is Involved in BMP-2 Signaling. J. Bone Miner. Res..

[48] Tang N., Song W., Luo J., Luo X., Chen J., Sharff K.A., Bi Y., He B., Huang J., Zhu G. (2008). BMP-9-induced osteogenic differentiation of mesenchymal progenitors requires functional canonical Wnt/β-catenin signalling. J. Cell. Mol. Med..

[49] Matsubara T., Kida K., Yamaguchi A., Hata K., Ichida F., Meguro H., Aburatani H., Nishimura R., Yoneda T. (2008). BMP2 Regulates Osterix through Msx2 and Runx2 during Osteoblast Differentiation. J. Biol. Chem..

[50] Phimphilai M., Zhao Z., Boules H., Roca H., Franceschi R.T. (2006). BMP signaling is required for RUNX2-dependent induction of the osteoblast phenotype. J. Bone Miner. Res..

[51] Chen F., Bi D., Cheng C., Ma S., Liu Y., Cheng K. (2018). Bone morphogenetic protein 7 enhances the osteogenic differentiation of human dermal-derived CD105+ fibroblast cells through the Smad and MAPK pathways. Int. J. Mol. Med..

[52] Myllylä R.M., Haapasaari K.-M., Lehenkari P., Tuukkanen J. (2013). Bone morphogenetic proteins 4 and 2/7 induce osteogenic differentiation of mouse skin derived fibroblast and dermal papilla cells. Cell Tissue Res..

[53] Zhu F., Friedman M.S., Luo W., Woolf P., Hankenson K.D. (2011). The transcription factor osterix (SP7) regulates BMP6-induced human osteoblast differentiation. J. Cell. Physiol..

[54] Jang W.-G., Kim E.-J., Kim D.-K., Ryoo H.-M., Lee K.-B., Kim S.-H., Choi H.-S., Koh J.-T. (2012). BMP2 Protein Regulates Osteocalcin Expression via Runx2-mediated Atf6 Gene Transcription. J. Biol. Chem..

[55] Yamaguchi A., Ishizuyaa T., Kintouab N., Wadaac Y., Katagirid T., Wozney J.M., Rosene V., Yoshikia S. (1996). Effects of BMP-2, BMP-4, and BMP-6 on Osteoblastic Differentiation of Bone Marrow-Derived Stromal Cell Lines, ST2 and MC3T3-G2/PA6. Biochem. Biophys. Res. Commun..

[56] Kim Y.-J., Lee M.-H., Wozney J.M., Cho J.-Y., Ryoo H.-M. (2004). Bone Morphogenetic Protein-2-induced Alkaline Phosphatase Expression Is Stimulated by Dlx5 and Repressed by Msx2. J. Biol. Chem..

[57] Li L., Jiang Y., Lin H., Shen H., Sohn J., Alexander P.G., Tuan R.S. (2019). Muscle injury promotes heterotopic ossification by stimulating local bone morphogenetic protein-7 production. J. Orthop. Transl..

[58] Dai G., Li Y., Liu J., Zhang C., Chen M., Lu P., Rui Y. (2020). Higher BMP Expression in Tendon Stem/Progenitor Cells Contributes to the Increased Heterotopic Ossification in Achilles Tendon With Aging. Front. Cell Dev. Biol..

[59] Jane J.A., Dunford B.A., Kron A., Pittman D.D., Sasaki T., Li J.Z., Li H., Alden T.D., Dayoub H., Hankins G.R. (2002). Ectopic Osteogenesis Using Adenoviral Bone Morphogenetic Protein (BMP)-4 and BMP-6 Gene Transfer. Mol. Ther..

[60] Leblanc E., Trensz F., Haroun S., Drouin G., Bergeron É., Penton C.M., Montanaro F., Roux S., Faucheux N., Grenier G. (2010). BMP-9-induced muscle heterotopic ossification requires changes to the skeletal muscle microenvironment. J. Bone Miner. Res..

[61] Prados B., del Toro R., MacGrogan D., Gómez-Apiñániz P., Papoutsi T., Muñoz-Cánoves P., Méndez-Ferrer S., de la Pompa J.L. (2021). Heterotopic ossification in mice overexpressing Bmp2 in Tie2+ lineages. Cell Death Dis..

[62] Kan L., Hu M., Gomes W.A., Kessler J.A. (2004). Transgenic Mice Overexpressing BMP4 Develop a Fibrodysplasia Ossificans Progressiva (FOP)-Like Phenotype. Am. J. Pathol..

[63] Agarwal S., Loder S.J., Breuler C., Li J., Cholok D., Brownley C., Peterson J., Hsieh H.H., Drake J., Ranganathan K. (2017). Strategic Targeting of Multiple BMP Receptors Prevents Trauma-Induced Heterotopic Ossification. Mol. Ther..

[64] Strong A.L., Spreadborough P.J., Dey D., Yang P., Li S., Lee A., Haskins R.M., Grimm P.D., Kumar R., Bradley M.J. (2021). BMP Ligand Trap ALK3-Fc Attenuates Osteogenesis and Heterotopic Ossification in Blast-Related Lower Extremity Trauma. Stem Cells Dev..

[65] Hwang C., Pagani C.A., Das N., Marini S., Huber A.K., Xie L., Jimenez J., Brydges S., Lim W.K., Nannuru K.C. (2020). Activin A does not drive post-traumatic heterotopic ossification. Bone.

[66] Hsieh H.H.S., Agarwal S., Cholok D.J., Loder S.J., Kaneko K., Huber A., Chung M.T., Ranganathan K., Habbouche J., Li J. (2019). Coordinating Tissue Regeneration Through Transforming Growth Factor-β Activated Kinase 1 Inactivation and Reactivation. Stem Cells.

[67] Strong A.L., Spreadborough P.J., Pagani C.A., Haskins R.M., Dey D., Grimm P.D., Kaneko K., Marini S., Huber A.K., Hwang C. (2020). Small molecule inhibition of non-canonical (TAK1-mediated) BMP signaling results in reduced chondrogenic ossification and heterotopic ossification in a rat model of blast-associated combat-related lower limb trauma. Bone.

[68] Shim J.-H., Greenblatt M.B., Xie M., Schneider M.D., Zou W., Zhai B., Gygi S., Glimcher L.H. (2009). TAK1 is an essential regulator of BMP signalling in cartilage. EMBO J..

[69] Zhang Y.E. (2009). Non-Smad pathways in TGF-β signaling. Cell Res..

[70] Gao L., Sheu T.-J., Dong Y., Hoak D.M., Zuscik M.J., Schwarz E.M., Hilton M.J., O’keefe R.J., Jonason J.H. (2013). TAK1 regulates SOX9 expression in chondrocytes and is essential for postnatal development of the growth plate and articular cartilages. J. Cell Sci..

[71] Onodera Y., Teramura T., Takehara T., Fukuda K. (2019). Transforming Growth Factor β-Activated Kinase 1 Regulates Mesenchymal Stem Cell Proliferation Through Stabilization of Yap1/Taz Proteins. Stem Cells.

[72] Cong Q., Liu Y., Zhou T., Zhou Y., Xu R., Cheng C., Chung H.S., Yan M., Zhou H., Liao Z. (2021). A self-amplifying loop of YAP and SHH drives formation and expansion of heterotopic ossification. Sci. Transl. Med..

[73] Khan F., Yu X., Hsiao E.C. (2021). Cardiopulmonary and Neurologic Dysfunctions in FibrodysplasiaOssificans Progressiva. Biomedicines.

[74] Valer J.A., Sánchez-De-Diego C., Pimenta-Lopes C., Rosa J.L., Ventura F. (2019). ACVR1 Function in Health and Disease. Cells.

[75] Wang T., Donahoe P.K. (2004). The immunophilin FKBP12: A molecular guardian of the TGF-beta family type I receptors. Front. Biosci..

[76] Chen Y.-G., Liu F., Massagué J. (1997). Mechanism of TGFβ receptor inhibition by FKBP12. EMBO J..

[77] Wang T., Li B.-Y., Danielson P.D., Shah P.C., Rockwell S., Lechleider R.J., Martin J., Manganaro T., Donahoe P.K. (1996). The Immunophilin FKBP12 Functions as a Common Inhibitor of the TGFβ Family Type I Receptors. Cell.

[78] Komatsu Y., Scott G., Nagy A., Kaartinen V., Mishina Y. (2006). BMP type I receptor ALK2 is essential for proper patterning at late gastrulation during mouse embryogenesis. Dev. Dyn..

[79] Yu X., Ton A.N., Niu Z., Morales B.M., Chen J., Braz J., Lai M.H., Barruet E., Liu H., Cheung K. (2022). ACVR1-activating mutation causes neuropathic pain and sensory neuron hyperexcitability in humans. Pain.

[80] Barruet E., Morales B.M., Cain C.J., Ton A.N., Wentworth K.L., Chan T.V., Moody T.A., Haks M.C., Ottenhoff T.H., Hellman J. (2018). NF-κB/MAPK activation underlies ACVR1-mediated inflammation in human heterotopic ossification. J. Clin. Investig..

[81] Rigueur D., Brugger S., Anbarchian T., Kil Kim J., Lee Y., Lyons K.M. (2014). The Type I BMP Receptor ACVR1/ALK2 is Required for Chondrogenesis During Development. J. Bone Miner. Res..

[82] Culbert A.L., Chakkalakal S.A., Theosmy E.G., Brennan T.A., Kaplan F.S., Shore E.M. (2014). Alk2 Regulates Early Chondrogenic Fate in Fibrodysplasia Ossificans Progressiva Heterotopic Endochondral Ossification. Stem Cells.

[83] Luo J., Tang M., Huang J., He B.-C., Gao J.-L., Chen L., Zuo G.-W., Zhang W., Luo Q., Shi Q. (2010). TGFβ/BMP Type I Receptors ALK1 and ALK2 Are Essential for BMP9-induced Osteogenic Signaling in Mesenchymal Stem Cells. J. Biol. Chem..

[84] Kaplan F.S., Xu M., Seemann P., Connor J.M., Glaser D.L., Carroll L., Delai P., Fastnacht-Urban E., Forman S.J., Gillessen-Kaesbach G. (2008). Classic and atypical fibrodysplasia ossificans progressiva (FOP) phenotypes are caused by mutations in the bone morphogenetic protein (BMP) type I receptor ACVR1. Hum. Mutat..

[85] Groppe J.C., Wu J., Shore E.M., Kaplan F.S. (2011). In vitro Analyses of the Dysregulated R206H ALK2 Kinase-FKBP12 Interaction Associated with Heterotopic Ossification in FOP. Cells Tissues Organs.

[86] Hatsell S.J., Idone V., Wolken D.M.A., Huang L., Kim H.J., Wang L., Wen X., Nannuru K.C., Jimenez J., Xie L. (2015). *ACVR1 ^R206H^* receptor mutation causes fibrodysplasia ossificans progressiva by imparting responsiveness to activin A. Sci. Transl. Med..

[87] Hino K., Ikeya M., Horigome K., Matsumoto Y., Ebise H., Nishio M., Sekiguchi K., Shibata M., Nagata S., Matsuda S. (2015). Neofunction of ACVR1 in fibrodysplasia ossificans progressiva. Proc. Natl. Acad. Sci. USA.

[88] Shimono K., Tung W.-E., Macolino C., Chi A.H.-T., Didizian J.H., Mundy C., A Chandraratna R., Mishina Y., Enomoto-Iwamoto M., Pacifici M. (2011). Potent inhibition of heterotopic ossification by nuclear retinoic acid receptor-γ agonists. Nat. Med..

[89] Pignolo R.J., Bedford-Gay C., Liljesthröm M., Durbin-Johnson B.P., Shore E.M., Rocke D.M., Kaplan F.S. (2015). The Natural History of Flare-Ups in Fibrodysplasia Ossificans Progressiva (FOP): A Comprehensive Global Assessment. J. Bone Miner. Res..

[90] Matsuo K., Chavez R.D., Barruet E., Hsiao E.C. (2019). Inflammation in Fibrodysplasia Ossificans Progressiva and Other Forms of Heterotopic Ossification. Curr. Osteoporos. Rep..

[91] Convente M.R., A Chakkalakal S., Yang E., Caron R.J., Zhang D., Kambayashi T., Kaplan F.S., Shore E.M. (2017). Depletion of Mast Cells and Macrophages Impairs Heterotopic Ossification in an *Acvr1R206H* Mouse Model of Fibrodysplasia Ossificans Progressiva. J. Bone Miner. Res..

[92] Matsuo K., Matsuo K., Lepinski A., Lepinski A., Chavez R.D., Chavez R.D., Barruet E., Barruet E., Pereira A., Pereira A. (2021). ACVR1R206H extends inflammatory responses in human induced pluripotent stem cell-derived macrophages. Bone.

[93] Ye Z., Wang S., Shan C., Zhu Q., Xue Y., Zhang K. (2023). The serum levels of activin A and bone morphogenetic protein-4 and -6 in patients with fibrodysplasia ossificans progressiva. Orphanet J. Rare Dis..

[94] Lees-Shepard J.B., Stoessel S.J., Chandler J.T., Bouchard K., Bento P., Apuzzo L.N., Devarakonda P.M., Hunter J.W., Goldhamer D.J. (2022). An anti-ACVR1 antibody exacerbates heterotopic ossification by fibro-adipogenic progenitors in fibrodysplasia ossificans progressiva mice. J. Clin. Investig..

[95] Yamamoto M., Stoessel S.J., Yamamoto S., Goldhamer D.J. (2022). Overexpression of Wild-Type ACVR1 in Fibrodysplasia Ossificans Progressiva Mice Rescues Perinatal Lethality and Inhibits Heterotopic Ossification. J. Bone Miner. Res..

[96] Williams E., Bagarova J., Kerr G., Xia D.-D., Place E.S., Dey D., Shen Y., Bocobo G.A., Mohedas A.H., Huang X. (2021). Saracatinib is an efficacious clinical candidate for fibrodysplasia ossificans progressiva. J. Clin. Investig..

[97] Pacifici M., Shore E.M. (2015). Common mutations in ALK2/ACVR1, a multi-faceted receptor, have roles in distinct pediatric musculoskeletal and neural orphan disorders. Cytokine Growth Factor Rev..

[98] Bloise E., Ciarmela P., Cruz C.D., Luisi S., Petraglia F., Reis F.M. (2019). Activin A in Mammalian Physiology. Physiol. Rev..

[99] Brown C.W., Li L., Houston-Hawkins D.E., Matzuk M.M. (2003). Activins Are Critical Modulators of Growth and Survival. Mol. Endocrinol..

[100] Morianos I., Papadopoulou G., Semitekolou M., Xanthou G. (2019). Activin-A in the regulation of immunity in health and disease. J. Autoimmun..

[101] Mundy C., Yao L., Sinha S., Chung J., Rux D., Catheline S.E., Koyama E., Qin L., Pacifici M. (2021). Activin A promotes the development of acquired heterotopic ossification and is an effective target for disease attenuation in mice. Sci. Signal..

[102] Wang H., Lindborg C., Lounev V., Kim J.-H., McCarrick-Walmsley R., Xu M., Mangiavini L., Groppe J.C., Shore E.M., Schipani E. (2016). Cellular Hypoxia Promotes Heterotopic Ossification by Amplifying BMP Signaling. J. Bone Miner. Res..

[103] Maxwell P.H., Wiesener M.S., Chang G.-W., Clifford S.C., Vaux E.C., Cockman M.E., Wykoff C.C., Pugh C.W., Maher E.R., Ratcliffe P.J. (1999). The tumour suppressor protein VHL targets hypoxia-inducible factors for oxygen-dependent proteolysis. Nature.

[104] Huang L.E., Arany Z., Livingston D.M., Bunn H.F. (1996). Activation of Hypoxia-inducible Transcription Factor Depends Primarily upon Redox-sensitive Stabilization of Its α Subunit. J. Biol. Chem..

[105] Kallio P.J., Pongratz I., Gradin K., McGuire J., Poellinger L. (1997). Activation of hypoxia-inducible factor 1α: Posttranscriptional regulation and conformational change by recruitment of the Arnt transcription factor. Proc. Natl. Acad. Sci. USA.

[106] Agarwal S., Loder S., Brownley C., Cholok D., Mangiavini L., Li J., Breuler C., Sung H.H., Li S., Ranganathan K. (2016). Inhibition of Hif1α prevents both trauma-induced and genetic heterotopic ossification. Proc. Natl. Acad. Sci. USA.

[107] Hino K., Horigome K., Nishio M., Komura S., Nagata S., Zhao C., Jin Y., Kawakami K., Yamada Y., Ohta A. (2017). Activin-A enhances mTOR signaling to promote aberrant chondrogenesis in fibrodysplasia ossificans progressiva. J. Clin. Investig..

[108] Hudson C.C., Liu M., Chiang G.G., Otterness D.M., Loomis D.C., Kaper F., Giaccia A.J., Abraham R.T. (2002). Regulation of Hypoxia-Inducible Factor 1α Expression and Function by the Mammalian Target of Rapamycin. Mol. Cell. Biol..

[109] Land S.C., Tee A.R. (2007). Hypoxia-inducible Factor 1α Is Regulated by the Mammalian Target of Rapamycin (mTOR) via an mTOR Signaling Motif. J. Biol. Chem..

[110] Zhang X., Li M., Yin N., Zhang J. (2021). The Expression Regulation and Biological Function of Autotaxin. Cells.

[111] Qureshi A.T., Dey D., Sanders E.M., Seavey J.G., Tomasino A.M., Moss K., Wheatley B., Cholok D., Loder S., Li J. (2017). Inhibition of Mammalian Target of Rapamycin Signaling with Rapamycin Prevents Trauma-Induced Heterotopic Ossification. Am. J. Pathol..

[112] Lin L., Shen Q., Leng H., Duan X., Fu X., Yu C. (2011). Synergistic Inhibition of Endochondral Bone Formation by Silencing Hif1α and Runx2 in Trauma-induced Heterotopic Ossification. Mol. Ther..

[113] Hwang C., Marini S., Huber A.K., Stepien D.M., Sorkin M., Loder S., Pagani C.A., Li J., Visser N.D., Vasquez K. (2019). Mesenchymal VEGFA induces aberrant differentiation in heterotopic ossification. Bone Res..

[114] Li D., Jiang Y., He P., Li Y., Wu Y., Lei W., Liu N., de Bruijn J.D., Zhang H., Zhang H. (2023). Hypoxia Drives Material-Induced Heterotopic Bone Formation by Enhancing Osteoclastogenesis via M2/Lipid-Loaded Macrophage Axis. Adv. Sci..

[115] Wyndaele J.J. (2010). Heterotopic ossification following spinal cord injury. Spinal Cord.

[116] Franz S., Rust L., Heutehaus L., Rupp R., Schuld C., Weidner N. (2022). Impact of Heterotopic Ossification on Functional Recovery in Acute Spinal Cord Injury. Front. Cell. Neurosci..

[117] Mujtaba B., Taher A., Fiala M.J., Nassar S., Madewell J.E., Hanafy A.K., Aslam R. (2019). Heterotopic ossification: Radiological and pathological review. Radiol. Oncol..

[118] Meyers C., Lisiecki J., Miller S., Levin A., Fayad L., Ding C., Sono T., McCarthy E., Levi B., James A.W. (2019). Heterotopic Ossification: A Comprehensive Review. JBMR Plus.

[119] Edsberg L.E., Crowgey E.L., Osborn P.M., Wyffels J.T. (2017). A survey of proteomic biomarkers for heterotopic ossification in blood serum. J. Orthop. Surg. Res..

[120] Garland D.E., A Hanscom D., A Keenan M., Smith C., Moore T. (1985). Resection of heterotopic ossification in the adult with head trauma. J. Bone Jt. Surg..

[121] Charter R.A., Chai C.J., Kim S.K., Kim E.S. (1990). Serum alkaline phosphatase and inorganic phosphorus values in spinal cord injury patients with heterotopic ossification. Spinal Cord.

[122] Hammond S.P. (2009). Yes, You Can! A Guide to Self-Care for Persons with Spinal Cord Injury.

[123] Zakel J.C., Harrington A.L. (2020). Heterotopic Ossification After Spinal Cord Injury: Current Clinical Approaches. Curr. Phys. Med. Rehabilitation Rep..

[124] Wang Q., Zhang P., Li P., Song X., Hu H., Li X., Chen W., Wang X. (2018). Ultrasonography Monitoring of Trauma-Induced Heterotopic Ossification: Guidance for Rehabilitation Procedures. Front. Neurol..

[125] McKean D., Ather S., Gandhi A., Hubble T., Belci M., Tiberti S., Papanikitas J., Yanny S., King D., Hughes R. (2020). Pelvic MRI in spinal cord injury patients: Incidence of muscle signal change and early heterotopic ossification. Spinal Cord.

[126] Shore E.M., Xu M., Feldman G.J., A Fenstermacher D., Cho T.-J., Choi I.H., Connor J.M., Delai P., Glaser D.L., LeMerrer M. (2006). A recurrent mutation in the BMP type I receptor ACVR1 causes inherited and sporadic fibrodysplasia ossificans progressiva. Nat. Genet..

[127] Shen Q., Little S.C., Xu M., Haupt J., Ast C., Katagiri T., Mundlos S., Seemann P., Kaplan F.S., Mullins M.C. (2009). The fibrodysplasia ossificans progressiva R206H ACVR1 mutation activates BMP-independent chondrogenesis and zebrafish embryo ventralization. J. Clin. Investig..

[128] Kaplan F.S., A Tabas J., Gannon F.H., Finkel G., Hahn G.V., A Zasloff M. (1993). The histopathology of fibrodysplasia ossificans progressiva. An endochondral process. J. Bone Jt. Surg..

[129] A Chakkalakal S., Zhang D., Culbert A.L., Convente M.R., Caron R.J., Wright A.C., DA Maidment A., Kaplan F.S., Shore E.M. (2012). An *Acvr1* R206H knock-in mouse has fibrodysplasia ossificans progressiva. J. Bone Miner. Res..

[130] Gannon F.H., Valentine B.A., Shore E.M., Zasloff M.A., Kaplan F.S. (1998). Acute Lymphocytic Infiltration in an Extremely Early Lesion of Fibrodysplasia Ossificans Progressiva. Clin. Orthop. Relat. Res..

[131] Davis T.A., Lazdun Y., Potter B.K., Forsberg J.A. (2013). Ectopic bone formation in severely combat-injured orthopedic patients — A hematopoietic niche. Bone.

[132] Lounev V.Y., Ramachandran R., Wosczyna M.N., Yamamoto M., DA Maidment A., Shore E.M., Glaser D.L., Goldhamer D.J., Kaplan F.S. (2009). Identification of Progenitor Cells That Contribute to Heterotopic Skeletogenesis. J. Bone Jt. Surg..

[133] Medici D., Shore E.M., Lounev V.Y., Kaplan F.S., Kalluri R., Olsen B.R. (2010). Conversion of vascular endothelial cells into multipotent stem-like cells. Nat. Med..

[134] Lin J., Yang Y., Zhou W., Dai C., Chen X., Xie Y., Han S., Liu H., Hu Y., Tang C. (2022). Single cell analysis reveals inhibition of angiogenesis attenuates the progression of heterotopic ossification in Mkx^−/−^ mice. Bone Res..

[135] Agarwal S., Loder S.J., Brownley C., Eboda O., Peterson J.R., Hayano S., Wu B., Zhao B., Kaartinen V., Wong V.C. (2015). BMP signaling mediated by constitutively active Activin type 1 receptor (ACVR1) results in ectopic bone formation localized to distal extremity joints. Dev. Biol..

[136] Barruet E., Garcia S.M., Wu J., Morales B.M., Tamaki S., Moody T., Pomerantz J.H., Hsiao E.C. (2021). Modeling the ACVR1R206H mutation in human skeletal muscle stem cells. eLife.

[137] Lees-Shepard J.B., Yamamoto M., Biswas A.A., Stoessel S.J., Nicholas S.-A.E., Cogswell C.A., Devarakonda P.M., Schneider M.J., Cummins S.M., Legendre N.P. (2018). Activin-dependent signaling in fibro/adipogenic progenitors causes fibrodysplasia ossificans progressiva. Nat. Commun..

[138] Dey D., Bagarova J., Hatsell S.J., Armstrong K.A., Huang L., Ermann J., Vonner A.J., Shen Y., Mohedas A.H., Lee A. (2016). Two tissue-resident progenitor lineages drive distinct phenotypes of heterotopic ossification. Sci. Transl. Med..

[139] Agarwal S., Loder S.J., Cholok D., Peterson J., Li J., Breuler C., Brownley R.C., Sung H.H., Chung M.T., Kamiya N. (2016). Scleraxis-Lineage Cells Contribute to Ectopic Bone Formation in Muscle and Tendon. Stem Cells.

[140] Yea J.-H., Gomez-Salazar M., Onggo S., Li Z., Thottappillil N., Cherief M., Negri S., Xing X., Qin Q., Tower R.J. (2023). Tppp3+ synovial/tendon sheath progenitor cells contribute to heterotopic bone after trauma. Bone Res..

[141] Pietras E.M., Warr M.R., Passegué E. (2011). Cell cycle regulation in hematopoietic stem cells. J. Cell Biol..

[142] Martinbianco E.M., Lilley C.M., Grech J., Mirza K.M., Chen X. (2022). Heterotopic Mesenteric Ossification With Trilineage Hematopoiesis. Cureus.

[143] Christofi T., A Raptis D., Kallis A., Ambasakoor F. (2008). True trilineage haematopoiesis in excised heterotopic ossification from a laparotomy scar: Report of a case and literature review. Ind. Mark. Manag..

[144] Borgia A., Manara S., Balzarotti M., Vinciguerra P., Di Maria A. (2020). Small lymphocytic lymphoma in true trilineage hematopoietic tissue within heterotopic ossification in an enucleated blind painful eye: A case report. J. Med Case Rep..

[145] Wang D., Shurafa M.S., Acharya D.R., Strand V.F., Linden M.D. (2004). Chronic Abdominal Pain Caused by Heterotopic Ossification With Functioning Bone Marrow: A Case Report and Review of the Literature. Arch. Pathol. Lab. Med..

[146] Jung Y., Song J., Shiozawa Y., Wang J., Wang Z., Williams B., Havens A., Schneider A., Ge C., Franceschi R.T. (2008). Hematopoietic Stem Cells Regulate Mesenchymal Stromal Cell Induction into Osteoblasts Thereby Participating in the Formation of the Stem Cell Niche. Stem Cells.

[147] Wilson A., Trumpp A. (2006). Bone-marrow haematopoietic-stem-cell niches. Nat. Rev. Immunol..

[148] Lucas T.S., Bab I.A., Lian J.B., Stein G.S., Jazrawi L., Majeska R.J., Attar-Namdar M., Einhorn T.A. (1997). Stimulation of Systemic Bone Formation Induced by Experimental Blood Loss. Clin. Orthop. Relat. Res..

[149] Otsuru S., Overholt K.M., Olson T.S., Hofmann T.J., Guess A.J., Velazquez V.M., Kaito T., Dominici M., Horwitz E.M. (2017). Hematopoietic derived cells do not contribute to osteogenesis as osteoblasts. Bone.

[150] Lee J.Y., Hong S.-H. (2020). Hematopoietic Stem Cells and Their Roles in Tissue Regeneration. Int. J. Stem Cells.

[151] Kawamoto H., Minato N. (2004). Myeloid cells. Int. J. Biochem. Cell Biol..

[152] Ebbo M., Crinier A., Vély F., Vivier E. (2017). Innate lymphoid cells: Major players in inflammatory diseases. Nat. Rev. Immunol..

[153] Gannon F.H., Glaser D., Caron R., Thompson L.D., Shore E.M., Kaplan F.S. (2001). Mast cell involvement in fibrodysplasia ossificans progressiva. Hum. Pathol..

[154] Glaser D.L., Economides A.N., Wang L., Liu X., Kimble R.D., Fandl J.P., Wilson J.M., Stahl N., Kaplan F.S., Shore E.M. (2003). In vivo somatic cell gene transfer of an engineered Noggin mutein prevents BMP4-induced heterotopic ossification. JBJS.

[155] Kaplan F.S., Shore E.M., Gupta R., Billings P.C., Glaser D.L., Pignolo R.J., Graf D., Kamoun M. (2005). Immunological Features of Fibrodysplasia Ossificans Progressiva and the Dysregulated BMP4 Pathway. Clin. Rev. Bone Miner. Metab..

[156] Kan L., Liu Y., McGuire T.L., Berger D.M.P., Awatramani R.B., Dymecki S.M., Kessler J.A. (2009). Dysregulation of Local Stem/Progenitor Cells as a Common Cellular Mechanism for Heterotopic Ossification. Stem Cells.

[157] Wosczyna M.N., A Biswas A., A Cogswell C., Goldhamer D.J. (2012). Multipotent progenitors resident in the skeletal muscle interstitium exhibit robust BMP-dependent osteogenic activity and mediate heterotopic ossification. J. Bone Miner. Res..

[158] Tirone M., Giovenzana A., Vallone A., Zordan P., Sormani M., Nicolosi P.A., Meneveri R., Gigliotti C.R., Spinelli A.E., Bocciardi R. (2019). Severe Heterotopic Ossification in the Skeletal Muscle and Endothelial Cells Recruitment to Chondrogenesis Are Enhanced by Monocyte/Macrophage Depletion. Front. Immunol..

[159] El-Labban N.G., Hopper C., Barber P. (1995). Ultrastructural finding of vascular degeneration in fibrodysplasia ossificans progressiva (FOP). J. Oral Pathol. Med..

[160] Qin Y., Guan J., Zhang C. (2014). Mesenchymal stem cells: Mechanisms and role in bone regeneration. Postgrad. Med. J..

[161] Knight M.N., Hankenson K.D. (2013). Mesenchymal Stem Cells in Bone Regeneration. Adv. Wound Care.

[162] Billings P.C., Fiori J.L., Bentwood J.L., O’Connell M.P., Jiao X., Nussbaum B., Caron R.J., Shore E.M., Kaplan F.S. (2008). Dysregulated BMP Signaling and Enhanced Osteogenic Differentiation of Connective Tissue Progenitor Cells From Patients With Fibrodysplasia Ossificans Progressiva (FOP). J. Bone Miner. Res..

[163] Yamaguchi A., Katagiri T., Ikeda T., Wozney J.M., Rosen V., A Wang E., Kahn A.J., Suda T., Yoshiki S. (1991). Recombinant human bone morphogenetic protein-2 stimulates osteoblastic maturation and inhibits myogenic differentiation in vitro. J. Cell Biol..

[164] Wang Y.X., Rudnicki M.A. (2012). Satellite cells, the engines of muscle repair. Nat. Rev. Mol. Cell Biol..

[165] McCarthy J.J., Mula J., Miyazaki M., Erfani R., Garrison K., Farooqui A.B., Srikuea R., Lawson B.A., Grimes B., Keller C. (2011). Effective fiber hypertrophy in satellite cell-depleted skeletal muscle. Development.

[166] Katagiri T., Yamaguchi A., Komaki M., Abe E., Takahashi N., Ikeda T., Rosen V., Wozney J.M., Fujisawa-Sehara A., Suda T. (1994). Bone morphogenetic protein-2 converts the differentiation pathway of C2C12 myoblasts into the osteoblast lineage [published erratum appears in J Cell Biol 1995 Feb;128(4):following 713]. J. Cell Biol..

[167] Hashimoto N., Kiyono T., Wada M.R., Umeda R., Goto Y.-I., Nonaka I., Shimizu S., Yasumoto S., Inagawa-Ogashiwa M. (2008). Osteogenic properties of human myogenic progenitor cells. Mech. Dev..

[168] Wang H., Zhang Q., Kaplan F.S., Pignolo R.J. (2021). Clearance of Senescent Cells From Injured Muscle Abrogates Heterotopic Ossification in Mouse Models of Fibrodysplasia Ossificans Progressiva. J. Bone Miner. Res..

[169] Wosczyna M.N., Konishi C.T., Carbajal E.E.P., Wang T.T., Walsh R.A., Gan Q., Wagner M.W., Rando T.A. (2019). Mesenchymal Stromal Cells Are Required for Regeneration and Homeostatic Maintenance of Skeletal Muscle. Cell Rep..

[170] Joe A.W., Yi L., Natarajan A., Le Grand F., So L., Wang J., Rudnicki M.A., Rossi F. (2010). Muscle injury activates resident fibro/adipogenic progenitors that facilitate myogenesis. Nat. Cell Biol..

[171] Uezumi A., Fukada S.-I., Yamamoto N., Takeda S., Tsuchida K. (2010). Mesenchymal progenitors distinct from satellite cells contribute to ectopic fat cell formation in skeletal muscle. Nature.

[172] Stanley A., Tichy E.D., Kocan J., Roberts D.W., Shore E.M., Mourkioti F. (2022). Dynamics of skeletal muscle-resident stem cells during myogenesis in fibrodysplasia ossificans progressiva. npj Regen. Med..

[173] Levesque J.-P., A Sims N., Pettit A.R., A Alexander K., Tseng H.-W., Torossian F., Genêt F., Lataillade J.-J., Le Bousse-Kerdilès M.-C. (2017). Macrophages Driving Heterotopic Ossification: Convergence of Genetically-Driven and Trauma-Driven Mechanisms. J. Bone Miner. Res..

[174] Bi Y., Ehirchiou D., Kilts T.M., A Inkson C., Embree M.C., Sonoyama W., Li L., I Leet A., Seo B.-M., Zhang L. (2007). Identification of tendon stem/progenitor cells and the role of the extracellular matrix in their niche. Nat. Med..

[175] Udagawa N. (2003). The mechanism of osteoclast differentiation from macrophages: Possible roles of T lymphocytes in osteoclastogenesis. J. Bone Miner. Metab..

[176] Vi L., Baht G.S., Whetstone H., Ng A., Wei Q., Poon R., Mylvaganam S., Grynpas M., A Alman B. (2014). Macrophages Promote Osteoblastic Differentiation In Vivo: Implications in Fracture Repair and Bone Homeostasis. J. Bone Miner. Res..

[177] Raggatt L.J., Wullschleger M.E., Alexander K.A., Wu A.C.K., Millard S.M., Kaur S., Maugham M.L., Gregory L.S., Steck R., Pettit A.R. (2014). Fracture Healing via Periosteal Callus Formation Requires Macrophages for Both Initiation and Progression of Early Endochondral Ossification. Am. J. Pathol..

[178] Wolken D.M.A., Idone V., Hatsell S.J., Yu P.B., Economides A.N. (2017). The obligatory role of Activin A in the formation of heterotopic bone in Fibrodysplasia Ossificans Progressiva. Bone.

[179] Shafritz A.B., Shore E.M., Gannon F.H., Zasloff M.A., Taub R., Muenke M., Kaplan F.S. (1996). Overexpression of an Osteogenic Morphogen in Fibrodysplasia Ossificans Progressiva. N. Engl. J. Med..

[180] Kaplan F.S., Glaser D.L., Shore E.M., Pignolo R.J., Xu M., Zhang Y., Senitzer D., Forman S.J., Emerson S.G. (2007). Hematopoietic Stem-Cell Contribution to Ectopic Skeletogenesis. J. Bone Jt. Surg..

[181] Champagne C.M., Takebe J., Offenbacher S., Cooper L.F. (2002). Macrophage cell lines produce osteoinductive signals that include bone morphogenetic protein-2. Bone.

[182] Del Zotto G., Antonini F., Azzari I., Ortolani C., Tripodi G., Giacopelli F., Cappato S., Moretta L., Ravazzolo R., Bocciardi R. (2017). Peripheral Blood Mononuclear Cell Immunophenotyping in Fibrodysplasia Ossificans Progressiva Patients: Evidence for Monocyte DNAM1 Up-regulation. Cytom. Part B Clin. Cytom..

[183] Maekawa H., Jin Y., Nishio M., Kawai S., Nagata S., Kamakura T., Yoshitomi H., Niwa A., Saito M.K., Matsuda S. (2022). Recapitulation of pro-inflammatory signature of monocytes with ACVR1A mutation using FOP patient-derived iPSCs. Orphanet J. Rare Dis..

[184] Evans K.N., Forsberg J.A., Potter B.K., Hawksworth J.S., Brown T.S., Andersen R., Dunne J.R., Tadaki D., Elster E.A. (2012). Inflammatory Cytokine and Chemokine Expression is Associated With Heterotopic Ossification in High-Energy Penetrating War Injuries. J. Orthop. Trauma.

[185] Forsberg J.A., Potter B.K., Polfer E.M., Safford S.D., Elster E.A. (2014). Do Inflammatory Markers Portend Heterotopic Ossification and Wound Failure in Combat Wounds?. Clin. Orthop. Relat. Res..

[186] Sorkin M., Huber A.K., Hwang C., Carson W.F., Menon R., Li J., Vasquez K., Pagani C., Patel N., Li S. (2020). Regulation of heterotopic ossification by monocytes in a mouse model of aberrant wound healing. Nat. Commun..

[187] Nunez J.H., Juan C.B., Sun Y., Hong J.B., Bancroft A.C.B., Hwang C., Medrano J.M., Huber A.K., Tower R.J., Levi B. (2023). Neutrophil and NETosis Modulation in Traumatic Heterotopic Ossification. Ann. Surg..

[188] Huang J., Wu J., Lin J., Li C., Tang B., Xiao H. (2022). Palovarotene inhibits the NF-κB signalling pathway to prevent heterotopic ossification. Clin. Exp. Pharmacol. Physiol..

[189] Kaplan F.S., Le Merrer M., Glaser D.L., Pignolo R.J., Goldsby R.E., Kitterman J.A., Groppe J., Shore E.M. (2008). Fibrodysplasia ossificans progressiva. Best Pr. Res. Clin. Rheumatol..

[190] Peng K., Cheung K., Lee A., Sieberg C., Borsook D., Upadhyay J. (2019). Longitudinal Evaluation of Pain, Flare-Up, and Emotional Health in Fibrodysplasia Ossificans Progressiva: Analyses of the International FOP Registry. JBMR Plus.

[191] Agarwal S., Loder S., Levi B. (2017). Heterotopic Ossification Following Upper Extremity Injury. Hand Clin..

[192] Lee S., Hwang C., Marini S., Tower R.J., Qin Q., Negri S., Pagani C.A., Sun Y., Stepien D.M., Sorkin M. (2021). NGF-TrkA signaling dictates neural ingrowth and aberrant osteochondral differentiation after soft tissue trauma. Nat. Commun..

[193] Qin Q., Gomez-Salazar M., Cherief M., Pagani C.A., Lee S., Hwang C., Tower R.J., Onggo S., Sun Y., Piplani A. (2022). Neuron-to-vessel signaling is a required feature of aberrant stem cell commitment after soft tissue trauma. Bone Res..

[194] Tomlinson R.E., Li Z., Zhang Q., Goh B.C., Li Z., Thorek D.L., Rajbhandari L., Brushart T.M., Minichiello L., Zhou F. (2016). NGF-TrkA Signaling by Sensory Nerves Coordinates the Vascularization and Ossification of Developing Endochondral Bone. Cell Rep..

[195] Salisbury E., Rodenberg E., Sonnet C., Hipp J., Gannon F.H., Vadakkan T.J., Dickinson M.E., Olmsted-Davis E.A., Davis A.R. (2011). Sensory nerve induced inflammation contributes to heterotopic ossification. J. Cell. Biochem..

[196] Ozen S., Şenlikci H.B., Yemişci O. (2020). Post-stroke bilateral heterotopic ossification: An acute problem with long-lasting consequences. Jt. Dis. Relat. Surg..

[197] Pek C., Lim M., Yong R., Wong H. (2014). Neurogenic heterotopic ossification after a stroke: Diagnostic and radiological challenges. Singap. Med. J..

[198] Huang H., Cheng W.-X., Hu Y.-P., Chen J.-H., Zheng Z.-T., Zhang P. (2017). Relationship between heterotopic ossification and traumatic brain injury. J. Orthop. Transl..

[199] Anthonissen J., Steffen C.T., Hofmann A., Victor J. (2020). The pathogenesis of heterotopic ossification after traumatic brain injury. A review of current literature. Acta Orthop. Belg..

[200] Hofman M., Koopmans G., Kobbe P., Poeze M., Andruszkow H., Brink P.R.G., Pape H.-C. (2015). Improved Fracture Healing in Patients with Concomitant Traumatic Brain Injury: Proven or Not?. Mediat. Inflamm..

[201] Liu W., Chen W., Xie M., Chen C., Shao Z., Zhang Y., Zhao H., Song Q., Hu H., Xing X. (2023). Traumatic brain injury stimulates sympathetic tone-mediated bone marrow myelopoiesis to favor fracture healing. Signal Transduct. Target. Ther..

[202] Chalidis B., Stengel D., Giannoudis P.V. (2007). Early Excision and Late Excision of Heterotopic Ossification after Traumatic Brain Injury Are Equivalent: A Systematic Review of the Literature. J. Neurotrauma.

[203] Genêt F., Ruet A., Almangour W., Gatin L., Denormandie P., Schnitzler A. (2015). Beliefs relating to recurrence of heterotopic ossification following excision in patients with spinal cord injury: A review. Spinal Cord.

[204] Mertens M.G., Meert L., Struyf F., Schwank A., Meeus M. (2021). Exercise Therapy Is Effective for Improvement in Range of Motion, Function, and Pain in Patients With Frozen Shoulder: A Systematic Review and Meta-analysis. Arch. Phys. Med. Rehabil..

[205] Casavant A.M., Hastings H. (2006). Heterotopic Ossification about the Elbow: A Therapist’s Guide to Evaluation and Management. J. Hand Ther..

[206] Fijn R., Koorevaar R., Brouwers J. (2003). Prevention of heterotopic ossification after total hip replacement with NSAIDs. Pharm. Weekbl..

[207] Griffin S.M., Sims S.H., Karunakar M.A., Seymour R., Haines N. (2013). Heterotopic Ossification Rates After Acetabular Fracture Surgery Are Unchanged Without Indomethacin Prophylaxis. Clin. Orthop. Relat. Res..

[208] Anthony P., Keys H., Evarts C.M., Rubin P., Lush C. (1987). Prevention of heterotopic bone formation with early post operative irradiation in high risk patients undergoing total HIP arthroplasty: Comparison of 10.00 Gy VS 20.00 Gy schedules. Endocrine.

[209] I Vasileiadis G., I Sakellariou V., Kelekis A., Galanos A., Soucacos P.N., Papagelopoulos P.J., Babis G.C. (2010). Prevention of heterotopic ossification in cases of hypertrophic osteoarthritis submitted to total hip arthroplasty. Etidronate or Indomethacin?. J. Musculoskelet. Neuronal Interact..

[210] Brennan T.A., Lindborg C.M., Bergbauer C.R., Wang H., Kaplan F.S., Pignolo R.J. (2017). Mast cell inhibition as a therapeutic approach in fibrodysplasia ossificans progressiva (FOP). Bone.

[211] Kaplan F.S., Shore E.M., Pignolo R.J.e., Hsiao E.C. (2011). The Medical Management of Fibrodysplasia Ossificans Progressiva: Current Treatment Considerations. Clinc. Proce. Intl. Clin. Consort. FOP.

[212] Wu X.-B., Yang M.-H., Zhu S.-W., Cao Q.-Y., Wu H.-H., Wang M.-Y., Cuellar D.O., Mauffrey C. (2014). Surgical resection of severe heterotopic ossification after open reduction and internal fixation of acetabular fractures: A case series of 18 patients. Injury.

[213] Kornhaber R., Foster N., Edgar D., Visentin D., Ofir E., Haik J., Harats M. (2017). The development and impact of heterotopic ossification in burns: A review of four decades of research. Scars Burn. Heal..

[214] Eekhoff E.M.W., Netelenbos J.C., de Graaf P., Hoebink M., Bravenboer N., Micha D., Pals G., de Vries T.J., A Lammertsma A., Raijmakers P.G. (2017). Flare-Up After Maxillofacial Surgery in a Patient With Fibrodysplasia Ossificans Progressiva: An [^18^F]-NaF PET/CT Study and a Systematic Review. JBMR Plus.

[215] Pignolo R.J., Hsiao E.C., Al Mukaddam M., Baujat G., Berglund S.K., Brown M.A., Cheung A.M., De Cunto C., Delai P., Haga N. (2023). Reduction of New Heterotopic Ossification (HO) in the Open-Label, Phase 3 MOVE Trial of Palovarotene for Fibrodysplasia Ossificans Progressiva (FOP). J. Bone Miner. Res..

[216] Pignolo R.J., Baujat G., Hsiao E.C., Keen R., Wilson A., Packman J., Strahs A.L., Grogan D.R., Kaplan F.S. (2020). Palovarotene for Fibrodysplasia Ossificans Progressiva (FOP): Results of a Randomized, Placebo-Controlled, Double-Blind Phase 2 Trial. J. Bone Miner. Res..

[217] Vanhoutte F., Liang S., Ruddy M., Zhao A., Drewery T., Wang Y., DelGizzi R., Forleo-Neto E., Rajadhyaksha M., Herman G. (2020). Pharmacokinetics and Pharmacodynamics of Garetosmab (Anti-Activin A): Results From a First-in-Human Phase 1 Study. J. Clin. Pharmacol..

[218] Rocco M.D., Forleo-Neto E., Pignolo R., Keen R., Orcel P., Funck-Brentano T., Roux C., Kolta S., Madeo A., Bubbear J.S. (2023). Garetosmab, an inhibitor of activin A, reduces heterotopic ossification and flare-ups in adults with fibrodysplasia ossificans progressiva: A randomized, double-blind, placebo-controlled phase 2 trial. medRxiv.

[219] Maekawa H., Kawai S., Nishio M., Nagata S., Jin Y., Yoshitomi H., Matsuda S., Toguchida J. (2020). Prophylactic treatment of rapamycin ameliorates naturally developing and episode -induced heterotopic ossification in mice expressing human mutant ACVR1. Orphanet J. Rare Dis..

[220] Haviv R., Moshe V., De Benedetti F., Prencipe G., Rabinowicz N., Uziel Y. (2019). Is fibrodysplasia ossificans progressiva an interleukin-1 driven auto-inflammatory syndrome?. Pediatr. Rheumatol..

[221] Nikishina I.P., Arsenyeva S.V., Matkava V.G., Arefieva A.N., Kaleda M.I., Smirnov A.V., Blank L.M., Kostik M.M. (2023). Successful experience of tofacitinib treatment in patients with Fibrodysplasia Ossificans Progressiva. Pediatr. Rheumatol..

[222] Tseng H.-W., Kulina I., Girard D., Gueguen J., Vaquette C., Salga M., Fleming W., Jose B., Millard S.M., Pettit A.R. (2020). Interleukin-1 Is Overexpressed in Injured Muscles Following Spinal Cord Injury and Promotes Neurogenic Heterotopic Ossification. J. Bone Miner. Res..

[223] Migliorini F., Trivellas A., Eschweiler J., Driessen A., Tingart M., Maffulli N. (2020). NSAIDs for Prophylaxis for Heterotopic Ossification After Total Hip Arthroplasty: A Bayesian Network Meta-analysis. Calcif. Tissue Int..

[224] Schneider J., Maffulli N., Eschweiler J., Bell A., Hildebrand F., Migliorini F. (2023). Efficacy of ibuprofen and indomethacin as prophylaxis of heterotopic ossification: A comparative study. Sci. Rep..

[225] Hoff P., Rakow A., Gaber T., Hahne M., Sentürk U., Strehl C., Fangradt M., Schmidt-Bleek K., Huscher D., Winkler T. (2013). Preoperative irradiation for the prevention of heterotopic ossification induces local inflammation in humans. Bone.

